# Targeting of SUMO substrates to a Cdc48–Ufd1–Npl4 segregase and STUbL pathway in fission yeast

**DOI:** 10.1038/ncomms9827

**Published:** 2015-11-05

**Authors:** Julie Bonne Køhler, Triin Tammsalu, Maria Mønster Jørgensen, Nana Steen, Ronald Thomas Hay, Geneviève Thon

**Affiliations:** 1 Department of Biology, University of Copenhagen, Ole MaalÃ¸es vej 5, Copenhagen DK-2200, Denmark; 2 Centre for Gene Regulation and Expression, Sir James Black Centre, College of Life Sciences, University of Dundee, Dow Street, Dundee DD1 5EH, UK

## Abstract

In eukaryotes, the conjugation of proteins to the small ubiquitin-like modifier (SUMO) regulates numerous cellular functions. A proportion of SUMO conjugates are targeted for degradation by SUMO-targeted ubiquitin ligases (STUbLs) and it has been proposed that the ubiquitin-selective chaperone Cdc48/p97-Ufd1-Npl4 facilitates this process. However, the extent to which the two pathways overlap, and how substrates are selected, remains unknown. Here we address these questions in fission yeast through proteome-wide analyses of SUMO modification sites. We identify over a thousand sumoylated lysines in a total of 468 proteins and quantify changes occurring in the SUMO modification status when the STUbL or Ufd1 pathways are compromised by mutations. The data suggest the coordinated processing of several classes of SUMO conjugates, many dynamically associated with centromeres or telomeres. They provide new insights into subnuclear organization and chromosome biology, and, altogether, constitute an extensive resource for the molecular characterization of SUMO function and dynamics.

## Abstract (web summary)

The small ubiquitin-like modifier SUMO can be conjugated to hundreds of protein species to affect their stability or activity. Here the authors use a quantitative proteomics approach to identify sumoylated proteins modified by the STUbL and Ufd1 pathways in fission yeast.

## Graphical abstract

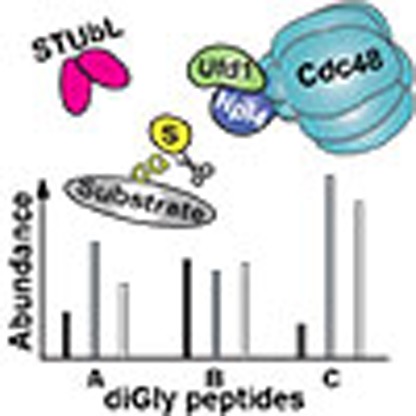

The small ubiquitin-like modifier (SUMO) can be conjugated to hundreds of protein species, following an evolutionarily conserved enzymatic cascade akin to ubiquitylation^1^. SUMO is thereby believed to participate in the regulation of multiple biological processes by changing the interaction properties of the proteins to which it is conjugated. Prior to conjugation, SUMO is processed to expose a conserved diglycine motif (diGly) at its carboxy terminus. Conjugation is then catalysed by the sequential action of an E1-activating, an E2-conjugating and an E3-ligating enzyme, which ultimately leads to the formation of an isopeptide bond between the carboxy terminus of SUMO and the ɛ amino-group of a lysine in the substrate. Self-modification of SUMO at internal lysine residues can furthermore result in the formation of polymeric SUMO chains^2^. The fission yeast sumoylating enzymes comprise the E1 heterodimer Rad31/Fub2 (Aos1/Uba2), the E2 enzyme Ubc9 (also known as Hus5) and two identified E3s of the SP-RING family; that is, the PIAS homolog Pli1 and the Mms21 homolog Nse2 (ref. 3). A fundamental aspect of sumoylation is its reversible nature. Desumoylation is carried out by SUMO-specific proteases (Ulp/SENP proteins) that by hydrolyzing SUMO isopeptide bonds allow proteins to return to their non-sumoylated form.

An alternative to desumoylation is the elimination of sumoylated proteins through the STUbL pathway. STUbLs, as represented by the Rfp1/Slx8 and Rfp2/Slx8 dimers in *Schizosaccharomyces pombe*, the Slx5/Slx8 dimer in *Saccharomyces cerevisiae*, and homo-dimeric RNF4 in mammals, are conserved ubiquitin E3 enzymes that target sumoylated proteins for ubiquitylation and subsequent degradation or recycling^4^,^5^,^6^,^7^,^8^. The activity of STUbLs is determined by a RING domain, commonly found in ubiquitin E3 ligases, and by several SUMO-interaction motifs (SIMs) that bind poly-sumoylated species^8^,^9^. High-molecular weight SUMO conjugates accumulate in STUbL-deficient cells and this phenotype is associated with genomic instability and general cellular dysfunction^5^,^7^,^10^. The best described STUbL substrate to date is the mammalian PML protein^8^,^11^. Other reported STUbL substrates include the transcriptional regulator Mot1 (ref. 12), the inner kinetochore protein CENP1 (ref. 13), the DNA damage protein MDC1 (refs 10, 14), the cohesin α-kleisin subunit Mcd1 (ref. 15) and the SUMO ligase Siz1 (ref. 16). Given the diversity of SUMO conjugates in the cell, other proteins are likely to be targeted by STUbLs under normal or perturbed conditions. Indeed, STUbLs have been detected at centromeres, nuclear pores, replication forks and at sites of DNA damage^17^,^18^.

A recently identified factor that also participates in the processing of sumoylated proteins is the conserved Cdc48–Ufd1–Npl4 segregase^19^,^20^,^21^. Cdc48–Ufd1–Npl4 has well-documented roles in several ubiquitin-related processes. By coupling the ubiquitin-binding properties of Ufd1–Npl4 with the ATPase activity of Cdc48 (p97 in mammals), Cdc48–Ufd1–Npl4 is believed to aid in the extraction of ubiquitylated proteins from higher order complexes^22^,^23^. Identification of a SIM motif in both fission and budding yeast Ufd1 revealed that Cdc48–Ufd1–Npl4 is in addition capable of recognizing sumoylated proteins, suggesting the complex might bind protein species co-modified by SUMO and ubiquitin, such as produced by STUbLs^20^,^21^. Like STUbL mutants, Ufd1 mutants also accumulate sumoylated proteins that coalesce into subnuclear foci^19^,^20^. Physical interactions between Cdc48, Ufd1 and STUbLs further support the idea that these proteins act in concert^19^,^21^. Cdc48–Ufd1–Npl4 may help mobilize STUbL substrates to promote their processing by the proteasome or by demodifying enzymes (Fig. 1a). Despite evidence suggesting a cooperative function, details of the substrates affected by a common STUbL/Cdc48–Ufd1–Npl4 pathway and the extent to which the two activities are coupled remain to be determined.

Mass spectrometry (MS)-based proteomics has become a widely used tool to study protein sumoylation. Hundreds of SUMO conjugates have been identified^24^,^25^,^26^,^27^,^28^, and, recently, techniques have been developed to map individual modification sites at a large scale^29^,^30^,^31^. This level of information is crucial to address the biological outcomes of individual sumoylation events. The identification of sumoylation sites has traditionally been hampered by an inability to enrich for SUMO modifications at the peptide level. Instead, protein-level purifications produce complex peptide mixtures from which site-specific information is difficult to extract. Moreover, the long SUMO branch left on modified peptides after trypsin cleavage gives rise to tandem MS (MS/MS) spectra that are difficult to interpret^32^. Here we use a newly designed two-step enrichment strategy that overcomes these obstacles and permits site-specific identification of SUMO targets at a proteome-wide scale^29^. In combination with Stable Isotope Labelling by Amino acids in Cell culture (SILAC)-based quantitative proteomics^33^, this strategy enabled us to identify >1,000 sumoylated lysines and to compare sumoylation levels for a subset of these sites between fission yeast wild-type and mutant strains deficient in STUbL (*slx8-1*) or Ufd1 (*ufd1ΔCt*^*213–342*^) function. These data suggest that certain substrates associated with centromeres and telomeres are coordinately processed by the combined action of the STUbL and Ufd1 pathways.

## Results

### Engineering of a His_6_SUMO^L109K^ variant for proteomic studies

**Figure 1:**
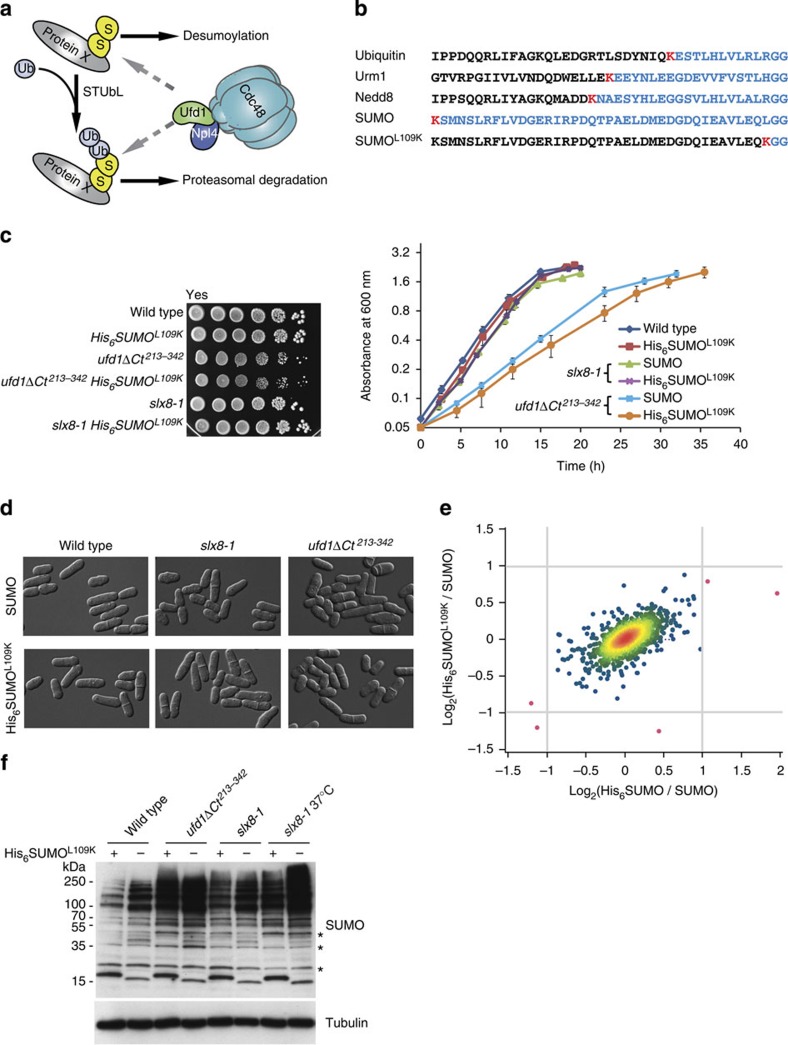
Strains engineered to test the effects of STUbLs and Cdc48–Ufd1–Npl4 on the sumoylated proteome of fission yeast. (**a**) Model for the processing of sumoylated species by STUbLs and Cdc48–Ufd1–Npl4. Sumoylated proteins can be targeted for degradation by STUbL-catalysed ubiquitylation. Cdc48–Ufd1–Npl4 may act directly on sumoylated proteins, or on proteins co-modified by SUMO and ubiquitin, to facilitate their recycling or proteasomal degradation. The strains shown in **b**–**f** were designed to identify substrates in these pathways. (**b**–**f**) Design and phenotypes of the His_6_SUMO^L109K^ variant. (**b**) Sequences of *S. pombe* ubiquitin-like proteins upstream of their carboxy-terminal diGly motifs. Residues highlighted in blue indicate the branch left on conjugates after endoproteinase Lys-C digestion. (**c**) Growth analysis of wild-type, *ufd1ΔCt*^*213–342*^ and *slx8-1* strains expressing SUMO or His_6_SUMO^L109K^. (**c**, left) Tenfold dilution series of the indicated strains were spotted onto rich medium (YES) and incubated at 33 °C for 3 days. From top to bottom: JK424, JK408, JK425, JK414, JK426 and JK418. (**c**, right) Growth curves. Cells were propagated at 30 °C in EMM2 minimal medium with required supplements and culture absorbance was recorded at 600 nm at the indicated time intervals. S.e. were computed from three independent cultures for each strain. (**d**) Micrographs of strains expressing SUMO or His_6_SUMO^L109K^. (**e**) 2D scatter plot showing the log_2_ relative abundance of 2,266 proteins between the samples His_6_SUMO/SUMO (*x*-axis) or His_6_SUMO^L109K^/SUMO (*y*-axis). Each point corresponds to a single protein and is coloured from green to red according to the density estimation value, reflecting the density of data points from less to more compressed. Proteins that are regulated by more than twofold are coloured in purple, however, none of these changes are statistically significant according to the multiple-sample analysis of variance test (Supplementary Data 1). (**f**) Western blot of wild-type SUMO and His_6_SUMO^L109K^ conjugates. Whole-cell extracts of the indicated strains were probed with a SUMO antibody. Tubulin was used as a loading control. Cultures were propagated at 30 °C unless indicated otherwise. Asterisks indicate cross-reactivity of the SUMO anti-serum.

We replaced the endogenous fission yeast SUMO gene (*pmt3*) with a His_6_-tagged version in which the leucine at position 109 is changed to a lysine (His_6_SUMO^L109K^ variant; Fig. 1b). A lysine at position 109 will, on digestion with the endoproteinase Lys-C, leave a diGly remnant on SUMO acceptor sites suitable for the purification of modified peptides with a diGly–Lys-specific antibody (anti-K-ɛ-GG)^29^. This antibody was originally prepared against ubiquitylated peptides for which a diGly remnant is left on trypsin digestion^34^. As ubiquitin contains an arginine at the position upstream its diGly sequence, Lys-C will not cleave at this site. Similarly, Lys-C digestion of proteins conjugated to other ubiquitin-like proteins generates long peptide-branches not recognized by the diGly–Lys antibody (Fig. 1b). Thus, Lys-C digestion of Ni_2_^+^-purified His_6_SUMO^L109K^ conjugates allows specific enrichment of SUMO diGly-modified peptides, facilitating MS analysis of the diGly-modified lysines based on their unique mass-to-charge ratio.

Functionality of the His_6_SUMO^L109K^ variant was verified by growth assays, microscopic examination, western blotting and MS. Cells expressing His_6_SUMO^L109K^ as their sole copy of SUMO, from the endogenous *pmt3* locus, displayed a wild-type morphology and growth rate (Fig. 1c,d). This is in sharp contrast to the growth defects of *pmt3Δ* cells. The proteome of strains expressing His_6_SUMO^L109K^ appeared unaltered compared with cells expressing wild-type SUMO (Fig. 1e and Supplementary Data 1). Also, *His*_*6*_*SUMO*^*L109K*^ cells responded to heat shock as wild-type, by a massive increase in high-molecular weight conjugates (Supplementary Fig. 1a). We combined *His*_*6*_*SUMO*^*L109K*^ with the s*lx8-1* STUbL mutation^5^ and with the *ufd1ΔCt*^*213–342*^ mutation truncating the C-terminal half of Ufd1 (ref. 19). There again, growth and morphological analyses found that *slx8-1* and *ufd1ΔCt*^*213–342*^ cells expressing His_6_SUMO^L109K^ were phenotypically similar to their wild-type SUMO counterparts at 30 °C (Fig. 1c,d). By western blotting, overall conjugation appeared slightly reduced for His_6_SUMO^L109K^ as previously observed for an analogous *S. cerevisiae* SUMO^I96K^ mutant^32^ (Fig. 1f). Reduced sumoylation has been shown to alleviate the growth defect of the *slx8-1* mutant at 36 °C (refs 5, 7) and indeed this was also the case with *slx8-1 His*_*6*_*SUMO*^*L109K*^ cells (Supplementary Fig. 1b). However, the sumoylation patterns detected in strains expressing His_6_SUMO^L109K^ or SUMO were globally very similar (Fig. 1f), in particular the changes in sumoylation occurring in the *slx8-1* and *ufd1ΔCt*^*213–342*^ mutants compared with wild type were conserved, pointing to the suitability of His_6_SUMO^L109K^ to globally map sumoylation sites of fission yeast wild-type and mutant cells.

### Proteome-wide identification of sumoylation sites

**Figure 2:**
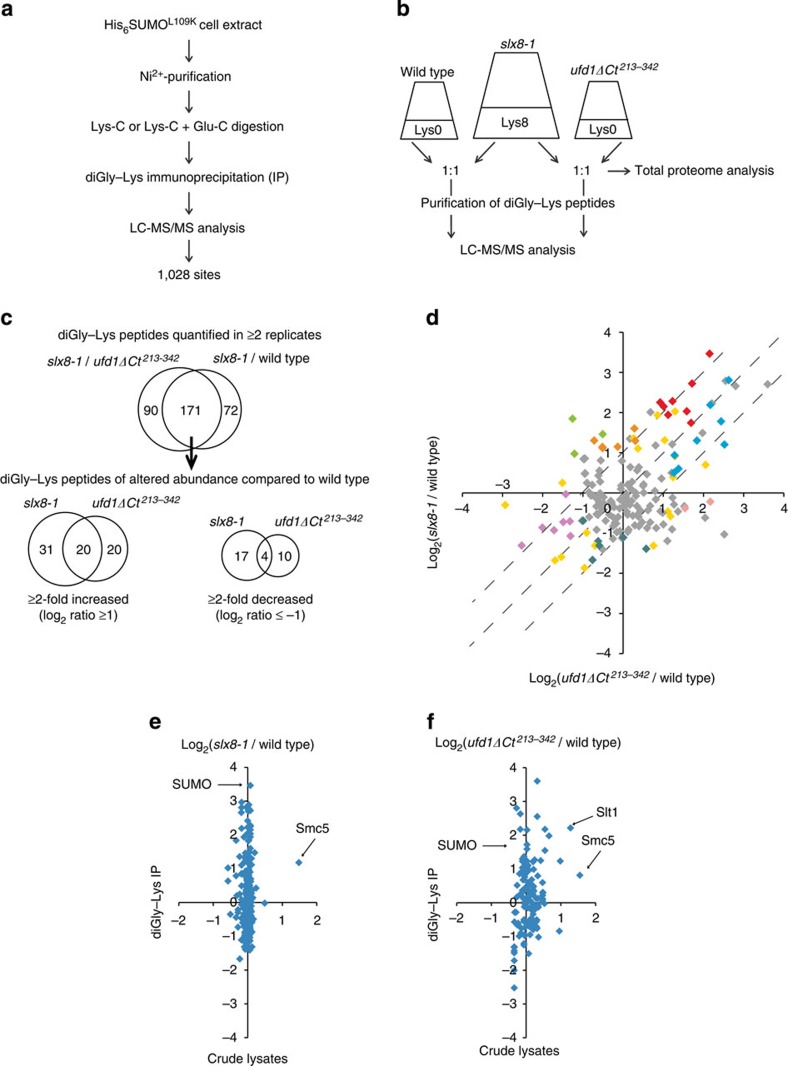
Quantitative identification of sumoylated residues in wild-type and *slx8-1* and *ufd1ΔCt*^*213–342*^ mutants. (**a**) Two-step enrichment for sumoylated peptides using *His*_*6*_*SUMO*^*L109K*^ strains. (**b**) Experimental design for SILAC experiments (see text for details). (**c**) Overview of peptides quantified in the triplicate SILAC experiments. Only diGly–Lys peptides for which SILAC ratios were obtained in at least two biological replicates were used in the analyses. In total, 171 distinct diGly–Lys peptides were quantified in both the *slx8-1*/ wild-type and *slx8-1*/*ufd1ΔCt*^*213–342*^ IPs, which enabled us to calculate *ufd1ΔCt*^*213–342*^/ wild-type ratios. See also Supplementary Fig. 2. (**d**) The normalized log_2_ ratios of quantified diGly–Lys-modified peptides for *ufd1ΔCt*^*213–342*^/ wild type and for *slx8-1*/wild type were plotted on a tsMAP, giving an overview of the relative change in abundance of individual peptides in each mutant. The two data sets correlate with a Pearson coefficient of *r*=0.49. Coloured data points represent peptides originating from the same proteins or from functionally related proteins, identified in Fig. 5. (**e**,**f**) Changes in protein sumoylation are not generally correlated with changes in protein concentration in either the *slx8-1* or *ufd1ΔCt*^*213–342*^ mutant. Among the proteins with quantified sumoylation sites, 112 and 79 were also quantified in the total proteome analyses of crude lysates for *slx8-1*/wild type and *ufd1ΔCt*^*213–342*^/wild type, respectively. Log_2_ SILAC ratios for sumoylated peptides were plotted against quantified ratios obtained for these proteins, yielding the maps in **e** and **f**.

To identify the sumoylated species that accumulate in Ufd1 mutants^19^,^20^, we isolated His_6_SUMO^L109K^-modified proteins from *ufd1ΔCt*^*213–342*^ cells. Cells were lysed under denaturing conditions and His_6_SUMO^L109K^ conjugates were isolated by Ni^2+^-chromatography. The purified material was digested with Lys-C, or Lys-C and Glu-C to generate shorter peptides, and diGly–Lys peptides were immunoprecipitated with anti-K-ɛ-GG antibody prior to MS analysis (Fig. 2a). This strategy identified a total of 841 unique diGly-modified lysines mapped with a localization probability of >0.75. Together these sumoylated sites defined a total of 414 proteins (Supplementary Data 2).

The His_6_SUMO^L109K^ variant was then employed in conjunction with SILAC-based proteomics^33^ to quantitatively compare the sumoylated proteomes of wild type and mutants defective in Ufd1 (*ufd1ΔCt*^*213–342*^) or STUbL (*slx8-1*). Two SILAC experiments were conducted in parallel, one experiment comparing a heavy lysine (Lys8) *slx8-1* culture with a light lysine (Lys0) wild-type culture, and the other experiment comparing the Lys8 *slx8-1* culture with a Lys0 *ufd1ΔCt*^*213–342*^ culture (Fig. 2b). The experiments were conducted in triplicate starting from isolated colonies, propagating cells at 30 °C and shifting them to 33 °C for the last 12 h to induce the *slx8-1* temperature-sensitive phenotype at the semi-permissive temperature of *slx8-1* (ref. 5). The *slx8-1* cultures were split, mixed separately in 1:1 ratios with wild-type and *ufd1ΔCt*^*213–342*^ cultures, and lysed. Small samples of cell lysate were subjected to in-solution digestion with Lys-C for total proteome analysis, while His_6_SUMO^L109K^-conjugated proteins and diGly-modified peptides were isolated from the remaining material. In total, the SILAC experiments detected 618 distinct sumoylation sites in 301 proteins. About 400 sites identified in the *ufd1ΔCt*^*213–342*^ strain had already been identified in the pilot experiment performed under non-SILAC conditions, indicating a high degree of experimental reproducibility (Supplementary Fig. 2). The higher complexity of SILAC peptide samples, where very intense SILAC pairs can hinder detection of less abundant species, might account for the fact that fewer sites were identified in the SILAC experiments than in the non-SILAC experiment even though each experiment used approximately the same amount of starting material. Together, the SILAC and non-SILAC experiments produced a comprehensive list of 1,028 sumoylation sites distributed between 468 proteins. This list is shown in Supplementary Data 2, the SILAC results are shown in Supplementary Data 3, and the total proteome analyses are shown in Supplementary Data 4. The lists comprise proteins ranging in abundance from very low to high according to the absolute quantifications in the study by Marguerat *et al*.^35^, indicating no experimental bias towards abundant species (Supplementary Fig. 3 and Supplementary Data 5).

### Comparing the effects of Ufd1 and STUbLs with SILAC

Among the diGly-modified peptides identified in the SILAC experiments, only peptides identified in both heavy and light forms in at least two biological replicates of either *slx8-1*/wild type or *slx8-1*/*ufd1ΔCt*^*213–342*^ were used for quantification. This resulted in the calculation of heavy-to-light ratios for 327 distinct peptides (Supplementary Data 3). For the total proteome samples, peptides quantified in at least one biological replicate lead to the quantification of 3,115 crude proteins (Supplementary Data 4). SILAC ratios for crude proteins were normalized using the MaxQuant software^36^, while the mean of their non-normalized ratios was used to normalize the ratios from the purified material^28^. Normalized SILAC ratios were used to derive median values for peptides measured in 3 biological replicates, or mean values for peptides quantified in only 2 biological replicates, resulting in the quantification of 243 and 261 diGly-modified peptides in the *slx8-1*/wild-type and *slx8-1*/*ufd1ΔCt*^*213–342*^ experiments, respectively (Supplementary Data 3). As 171 of these peptides were quantified for both *slx8-1*/wild type and *slx8-1*/*ufd1ΔCt*^*213–342*^ (Fig. 2c) their relative abundance in *ufd1ΔCt*^*213–342*^ and wild type could also be calculated. Figure 2d compares the *slx8-1*/wild-type and *ufd1ΔCt*^*213–342*^/wild-type ratios. Sumoylation was increased ⩾2-fold for 51 sites (in 33 proteins) in the *slx8-1* mutant and for 40 sites (in 31 proteins) in the *ufd1ΔCt*^*213–342*^ mutant. Conversely, sumoylation was reduced ⩾2-fold for 14 and 21 sites in the 2 mutants, respectively. Regression analysis showed the two data sets are correlated with a Pearson coefficient of *r*=0.49. Even with a great degree of variation, this supports the existence of common tendencies in sumoylation dynamics in the two strains.

### Comparison of sumoylation and total protein levels

Should proteins with increased sumoylation in *slx8-1* and/or *ufd1ΔCt*^*213–342*^ be normally targeted for degradation by STUbL-mediated ubiquitylation, these proteins may be stabilized in the mutant backgrounds. We could quantify protein levels for a subset of proteins with quantified sumoylation sites (112 and 79 proteins were quantified in both crude and diGly–Lys immunoprecipitated samples for *slx8-1*/wild type and *ufd1ΔCt*^*213–342*^/wild type, respectively). For these proteins, changes in sumoylation were not generally correlated with a change in protein abundance (Fig. 2e,f). Importantly, the abundance of SUMO itself was unchanged in the mutants excluding the possibility that protein sumoylation is increased in the *slx8-1* or *ufd1ΔCt*^*213–342*^ mutant due to elevated SUMO concentration. Two exceptions were Slt1, an uncharacterized orphan protein, which displayed a nearly twofold increase in *ufd1ΔCt*^*213–342*^ cells compared with a threefold increase in sumoylation, and Smc5, which increased at the protein level by nearly threefold in both mutants.

### General features of the SUMO proteome at the residue level

**Figure 3:**
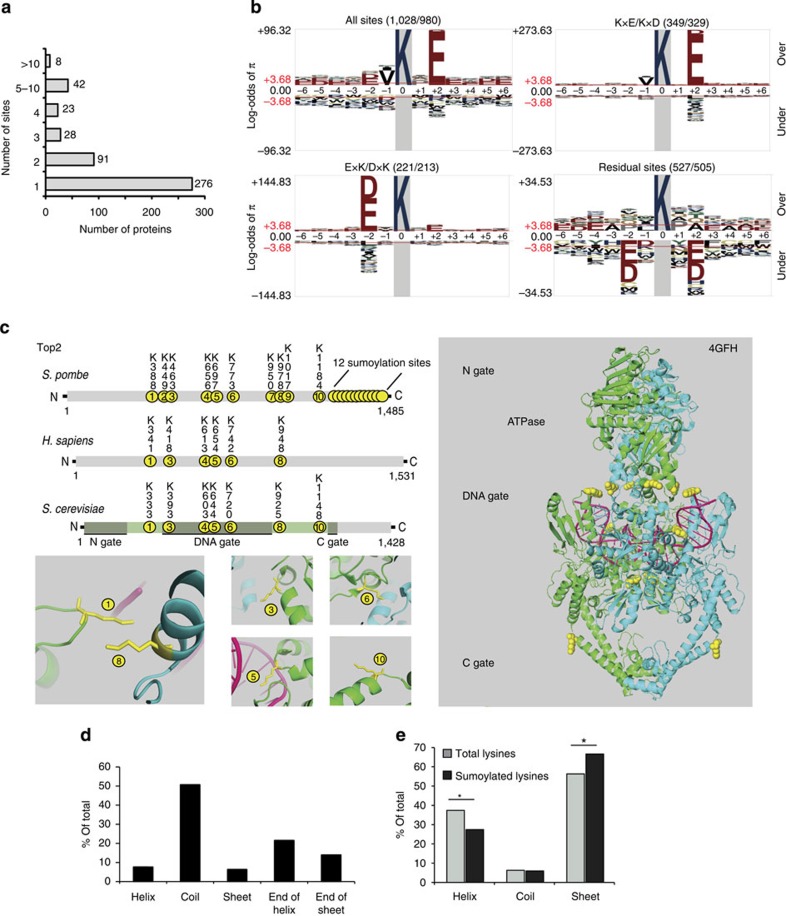
General features of sumoylation at the residue level. **a**) Distribution of sites between sumoylated proteins.(**b**) Amino-acid context of sumoylated lysines. Four pLogo graphs display the amino-acid sequence context of all sumoylated lysines; of KxE/KxD consensus-site lysines; of lysines within the ExK/DxK inverted consensus site and of sites not conforming to either consensus (residual sites). The numbers in parentheses above each graph indicate the total number of sites in the category (first number) and, among them, the number of sites that covered the full 13-amino acids window required for the pLogo analysis (second number). The log-odds of the binomial probability (*π*) are represented on the *y*-axis. Significance threshold values of 3.68 (*P*<0.05) are shown in red. (**c**) Sumoylated residues identified in this study are shown in yellow in a cartoon structure of *S. cerevisiae* topoisomerase 2 (PDB#4GFH; 94% identical to *S. pombe*). The portion of the protein whose structure was solved is coloured in the *S. cerevisiae* Top2 representation on the right. Enlarged cartoon views show the tendency of sumoylation sites to fall into coils or end of helices, the proximity of sumoylation sites in three-dimension and the proximity of sites to DNA. (**d**) Distribution of 82 sumoylated lysines identified in this study and mapped onto existing crystal structures using PyMOL. The structures are shown in Supplementary Figs 4 and 5 and the sites are listed in Supplementary Data 7. ‘End-of-helix' and ‘end-of-sheet' refer to lysines at three or less amino acids from the end of a helix or sheet. (**e**) Distribution of lysines in secondary structures predicted with PSIPRED^70^ for respective sumoylated lysines, and for all the lysines in proteins with a sumoylated lysine. *P<0.001. *P* values were calculated with a hypergeometric probability test. See also Supplementary Data 8.

As our study is the first global analysis of sumoylation in *S. pombe*, and one of the very first to identify sumoylation sites globally in any organism, it also provided important insights into sumoylation *per se*. Nearly half the proteins identified in our combined experiments displayed multiple sumoylation sites (Fig. 3a). Some proteins contained many sites with, for example, 14 sites identified in Uba2, 17 sites in the HMG-box protein Hmo1 or 22 sites in the topoisomerase Top2. We identified seven SUMO–SUMO branching points (K14, K30, K39, K51, K60, K63 and K71, with co-sumoylation of K14 and K30), and multiple sumoylation sites in ubiquitin (K6, K11, K27, K48 and K63). Doubly or triply modified peptides were detected for 22 proteins even though our approach detects co-modification only for sequential lysines, pointing to widespread multi-sumoylation. Altogether these observations are consistent with features of sumoylation recently proposed for mammalian cells^29^,^30^,^31^. Half of the sumoylation sites identified conformed to the forward (ΨKxD/E)^37^ or reversed (D/ExKΨ)^27^ consensus motif proposed in other organisms, while the remaining sites did not conform to any identifiable motif (Fig. 3b).

In addition to multiply sumoylated peptides, sumoylated peptides bearing other post-translational modifications were also detected (Supplementary Data 6), including branched SUMO peptides phosphorylated on T20 and T24. Co-modifications suggestive of cross-talks were observed for the histones H3, H2A and H2B. In particular, concomitant phosphorylation of S121 and sumoylation of K119 occurred on H2A-alpha and -beta. H2AS121 is phosphorylated by Bub1, mediator of the spindle assembly checkpoint^38^. H2AS121 phosphorylation might facilitate sumoylation at K119 by introducing a negative charge at the +2 position and effectively creating a consensus modification site.

To investigate the structural features of SUMO modification sites in proteins, we mapped sumoylation sites onto known protein structures. Of the 22 sites in Top2 (Fig. 3c), we mapped 10 to the DNA gate, with 6 at the interface of the ATPase domain and DNA-binding and cleavage core (Fig. 3c). One sumoylated lysine (K388 in *S. pombe* or K333 in *S. cerevisiae*) is in the K loop, whose interaction with DNA has been proposed to trigger ATP hydrolysis and release of DNA from Top2 (ref. 39). In addition, 12 sumoylation sites are in the last 302 amino acids of *S. pombe* Top2, in a part of the protein not present in the crystal structure but whose sumoylation is also conserved^29^,^30^,^40^ and likely to interfere with the functioning of the C gate. Supplementary Figure 4 displays sites in Cdc48 and in the nucleosome, and Supplementary Fig. 5 displays a total of 73 additional sites in 9 *S. pombe* proteins and 34 highly conserved proteins from other organisms (82 sites in total; Supplementary Data 7). Several conclusions could be reached from this analysis. First, nearly all sites were accessible at the surface of proteins indicating sumoylation occurs on folded proteins. Second, sites located in different domains of a protein could be very close in three-dimensional structures as for K388 and K978 in Top2, or in the case of the nucleosome where modification sites on distinct histones (H3K79 and H4K79) are close together in the nucleoprotein complex. Finally, sites were predominantly in loops or coils or at the very end of alpha helices or beta sheets (Fig. 3d). Consistently, a PSIPRED secondary structure prediction for the proteins in our list (Supplementary Data 2) found sumoylated lysines preferentially in coils, with an under-representation in helices (Fig. 3e and Supplementary Data 8).

### Functional clustering of sumoylated proteins

**Figure 4:**
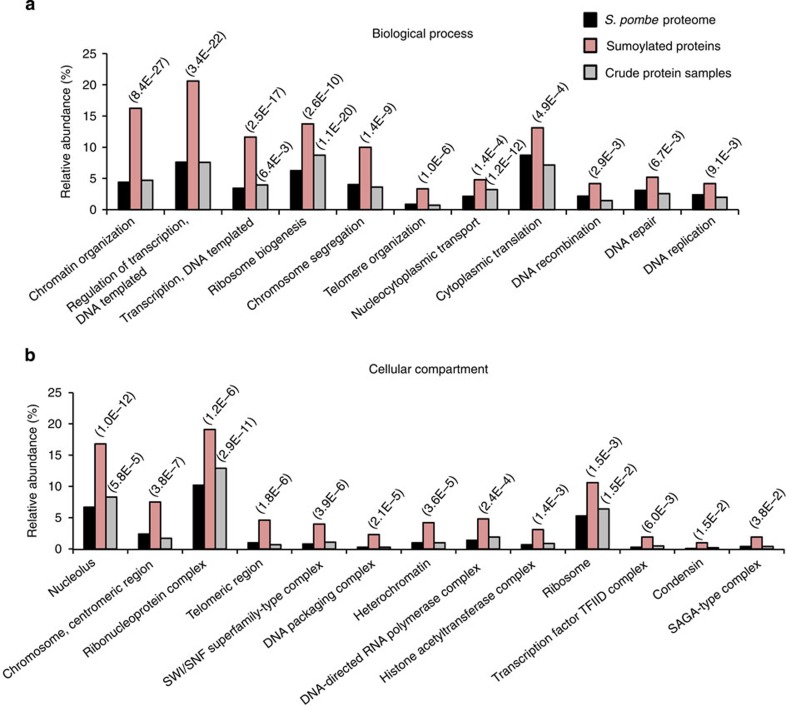
Functional analysis of the *S. pombe* sumoylated proteome. (**a**) Gene Ontology (GO)-slim ‘biological process' analysis on the ensemble of sumoylated proteins identified in all experiments combined. The bar graph shows some of the most significantly enriched ‘process terms' (*P*<0.01) in a comparison with the predicted *S. pombe* proteome. For comparison, the same analysis was performed on the proteins identified in crude lysates prior to SUMO enrichment. ‘Relative abundance' refers to the fraction of proteins belonging to a given GO-category in the indicated sample (that is, entire *S. pombe* proteome, sumoylated proteins identified in diGly–SUMO IP or crude lysates). The analysis was performed using the GOTermMapper online tool at Princeton, and *P* values were calculated with a hypergeometric probability test. (**b**) GO enrichment analysis of ‘cellular compartment' terms for sumoylated proteins using the AmiGo online tool. Selected enriched GO-categories (*P*<0.05) are arranged according to their degree of significance.

Gene ontology analyses of the ensemble of identified SUMO targets (Supplementary Data 2) found an over-representation of proteins participating in chromosome organization and segregation, transcription, as well as DNA recombination and repair (Fig. 4a). Also enriched were proteins associated with ribosome biogenesis and nucleo-cytoplasmic transport. These enrichments are not surprising given the literature on SUMO and the predominantly nuclear localization of SUMO in *S. pombe*^24^,^26^,^41^.

Corroborating another hallmark of sumoylation^24^,^26^,^42^, many of the *S. pombe* proteins targeted for sumoylation were part of heavily sumoylated macromolecular complexes (Supplementary Data 2 and Fig. 4b). For example, the SWI/SNF chromatin-remodelling complex, the histone acetylase SAGA complex and the TFIID transcription factor and RNA polymerase core complexes were sumoylated on multiple subunits. The nucleolar U3 sno-RNA-containing complex was sumoylated at 15 residues, on 9 subunits. Other complexes highly targeted by sumoylation were the core cohesion, SMC5/6, and condensin complexes, with the latter modified on all 5 components with a total of 17 sumoylation sites.

### Functional classes of conjugates targeted by STUbL or Ufd1

**Figure 5:**
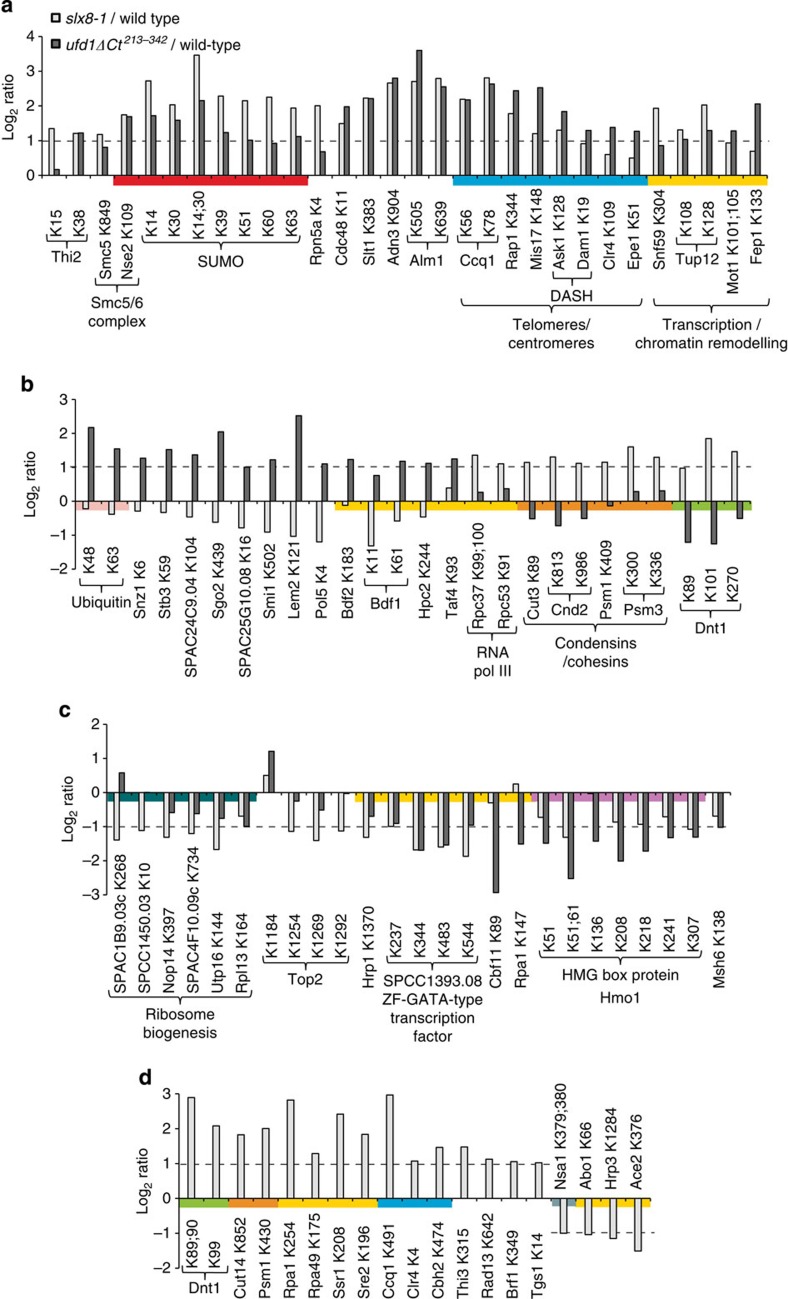
Sumoylated peptides with a ⩾2-fold change in abundance in the *slx8-1* or *ufd1ΔCt*^*213–342*^ mutant. Colours highlight peptides originating from the same proteins or from proteins with related function. The same peptides are coloured in the tsMAP in Fig. 2d. (**a**) Peptides of increased abundance in both mutants. (**b**) Peptides of increased abundance in single mutants. (**c**) Peptides of decreased abundance in either mutant. (**d**) Peptides quantified for only *slx8-1*/wild type.

The SILAC data sets (Supplementary Data 3) were analysed to identify cellular functions relying on the processing of SUMO conjugates by STUbLs or Cdc48–Npl4–Ufd1. The peptides whose sumoylation was most affected by the *slx8-1* or *ufd1ΔCt*^*213–342*^ mutations are displayed in bar graphs in Fig. 5. Peptides were loosely grouped in the graphs according to whether their sumoylation was increased in both mutants (Fig. 5a; the largest and most strongly affected class), increased specifically in one mutant (Fig. 5b) or decreased in either mutant (Fig. 5c). Peptides originating from the same proteins were placed side–by-side, revealing strongly correlated patterns of sumoylation within proteins. Some proteins contained sites whose sumoylation was below the cutoff used in Fig. 5; in general sumoylation at these sites changed with the same trends as for the peptides on display, just with a reduced amplitude (Supplementary Data 3). Peptides whose abundance was measured only in the *slx8-1*/wild-type SILAC experiment are shown in Fig. 5d. Several of these peptides originated from proteins represented in the upper graphs, in all cases corroborating the observed changes.

The function of the proteins whose sumoylation status strongly depends on Slx8 or Ufd1 was determined using the Pombase^43^ and Pubmed databases, allowing us to annotate the graphs in Fig. 5 accordingly. Protein localization data^43^ were also used, although not systematically displayed. Functions could be assigned for most proteins. Colour was added in both Fig. 5 and Fig. 2d to label peptides according to their functional groups.

**Figure 6:**
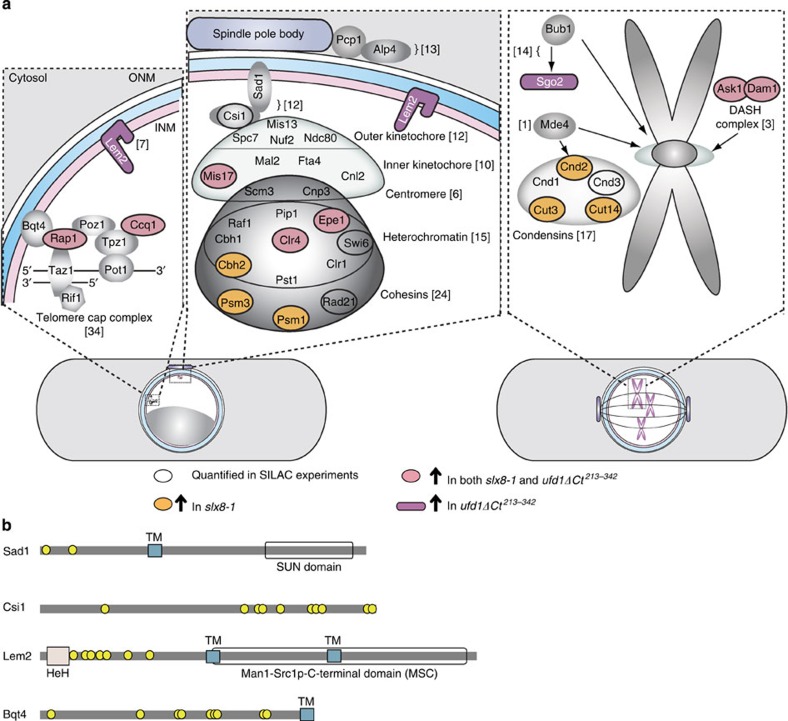
Sumoylated proteins targeted by Slx8 or Ufd1 at centromeres, telomeres and nuclear envelope. Only proteins for which sumoylation sites were identified in this study are represented. (**a**) Examples of interacting protein complexes with multiple sumoylated subunits. The number of sumoylation sites are indicated in brackets. *S. pombe* centromeres cluster at the spindle pole body (SPB) in interphase cells through inner and outer nuclear membrane (INM and ONM) proteins. Proteins associating with the centromeres and kinetochores of mitotic chromosomes are displayed on the right. The sumoylation of circled proteins was quantified in the SILAC experiments. Proteins with increased sumoylation specifically in *slx8-1*, specifically in *ufd1ΔCt*^*213–342*^ or in both mutant backgrounds are highlighted in orange, purple or pink, respectively. (**b**) Schematic representation of sumoylation sites identified in nuclear-envelope-associated proteins involved in centromere and/or telomere anchoring. Yellow circles specify sumoylated lysines. Transmembrane domains are indicated with blue squares. Sumoylation sites were exclusively detected in the nucleoplasmic parts of the proteins, and almost exclusively in the *ufd1ΔCt*^*213–342*^ mutant. HeH, helix–extension–helix domain; SUN, Sad1p UNC-84 domain).

Remarkable functional clustering was observed. More than a third of the proteins whose sumoylation coordinately increased in both mutants (7/20; Fig. 5a) are proteins associated with centromeres or telomeres. Figure 6 displays all the SUMO conjugates identified in our study localizing to these chromosomal regions and labels conjugates quantified in the SILAC experiments. In total, the sumoylation of 17 centromere- or telomere-associated proteins was quantified in both mutants. The sumoylation status of 13 of them was significantly increased in Ufd1 or Slx8 (Fig. 6a).

It was also notable that condensins and cohesins constituted more than half of the proteins whose sumoylation was specifically increased in the *slx8-1* mutant (Fig. 5b), representing nearly all condensins and cohesins measured in the SILAC experiments. The sumoylation state of numerous transcription factors and chromatin remodellers also changed in the mutants, but not in a unique pattern, consistent with the diversity of function and subnuclear compartimentalization of this class of proteins (Fig. 5). Finally, sumoylation of both SUMO and ubiquitin was affected in the mutants, *albeit* differently. SUMO branches increased greatly in both mutants (Fig. 5a). Sumoylation of ubiquitin increased specifically in the *ufd1ΔCt*^*213–342*^ mutant (Fig. 5b).

### Comparison with sumoylation in *S. cerevisiae*

We compared our *S. pombe* data (Supplementary Data 2) with five *S. cerevisiae* large-scale proteomic studies of sumoylation^24^,^25^,^44^,^45^,^46^ in YeastMine^47^, filtering for proteins that have orthologues in both species (Supplementary Fig. 6 and Supplementary Data 9). As noted previously^45^, the five studies are in poor concordance with each other. Comparing them individually with our more comprehensive data sets indicates the poor concordance is partly due to limited coverage. Merging four of the *S. cerevisiae* lists^24^,^25^,^44^,^45^ finds that sumoylation is conserved between the two species for at least 42% of the 412 *S. pombe* proteins listed in Supplementary Data 2 for which orthologues were found in *S. cerevisiae*.

The study by Albuquerque *et al*.^45^ is of particular interest since it identifies potential STUbL substrates in *S. cerevisiae*, using the *slx5Δ* mutant. Comparing with the potential substrates identified here for Slx8 reveals strong evolutionary conservation for some classes of substrates in particular cohesins, condensins and for the Smc5/6 complex (Supplementary Fig. 7). The kinetochore and DASH complex components identified here in the case of Slx8 were not identified for Slx5, but another kinetochore protein, CBF2, and regulators of kinetochore-microtubule attachment (SLI5 and BIR1) were identified, suggesting STUbLs and Cdc48–Ufd1–Npl4 might have conserved roles of in chromosome segregation. Divergence was observed for septins, a major class of proposed substrates for *S. cerevisiae* STUbL^45^, for which sumoylation appears much less prominent in *S. pombe*, possibly reflecting diverged modes of septation between fission and budding yeast.

## Discussion

Using a recently developed purification strategy, we have analysed the SUMO proteome of fission yeast wild-type and mutant cells, identifying over a thousand unique sumoylation sites in 468 different proteins in conditions where SUMO was expressed at endogenous levels. Our data sets highlight the participation of SUMO in higher order protein complexes and the widespread links between SUMO and chromosome transactions. They provide a quantitative overview of the effects of STUbLs and Ufd1 on the sumoylated proteome, showing that both STUbLs and Cdc48–Ufd1–Npl4 target discrete classes of sumoylated proteins and identifying potential substrates sequentially processed by STUbLs and Cdc48–Ufd1–Npl4. The modification status of SUMO itself, and of ubiquitin, suggest a sequential action of Slx8 and Ufd1 that would result in the accumulation of SUMO–SUMO linkages in both mutants, and of mixed ubiquitin–SUMO chains specifically in the Ufd1 mutant. Some of the protein groups affected individually or in a correlated manner by STUbLs and Ufd1 are discussed below.

Many potential targets of a STUbL/Ufd1 pathway were at centromeres/kinetochores, telomeres and at the nuclear envelope (NE) (Figs 5a and 6a). They include Mis17, Ask1 and Dam1. Mis17 associates with the inner kinetochore plate together with the Mis6–Mal2–Sim4 complex (CENP-I/-O/-K homologues) throughout the cell cycle^48^, while Ask1 and Dam1 are part of the DASH complex that binds the outer kinetochore only during mitosis, to facilitate the attachment of kinetochores to the mitotic spindle and subsequent chromosome segregation^49^,^50^. In human cells, RNF4-mediated turnover of the inner kinetochore homologue CENP-I has been proposed to help form the inner kinetochore plate^13^. Our finding of elevated Mis17 sumoylation in STUbL and Ufd1 mutant cells indicates a conserved role for STUbLs and Ufd1 in regulating inner kinetochore structures. STUbL/Ufd1-mediated turnover of outer kinetochore components may similarly contribute to normal spindle and chromosomes dynamics, possibly accounting for some of the mitotic defects observed in STUbL mutants^17^.

At telomeres, sumoylation of the shelterin proteins Rap1 and Ccq1 was increased in the three to sixfold range in both mutants (Fig. 5a,d). The shelterin complex protects the ends of chromosomes and regulates their replication by telomerase. In *S. cerevisiae*, non-functional poly-sumoylated versions of Rap1 are cleared by Uls1, a dual STUbL and DNA-dependent ATPase/translocase, to sustain Rap1-dependent inhibition of telomeric end-to-end fusions^51^. A similar function could be carried out by the combination of Slx8/Rfp STUbL and Cdc48–Ufd1–Npl4-dependent ATPase activity.

Not only were kinetochore and shelterin components heavily sumoylated in the *slx8-1* and *ufd1ΔCt*^*213–342*^ mutants, but their anchors at the nuclear periphery were also increasingly sumoylated in the Ufd1 mutant. In *S. pombe*, the association of telomeres with the NE relies on interactions between telomeric shelterin proteins (Taz1 and Rap1) and the inner nuclear membrane proteins Bqt3 and Bqt4 (refs 52, 53). During mitosis these associations are released to allow faithful chromosome segregation. Other factors implicated in telomere–NE anchoring include the integral nuclear membrane proteins of the animal LEM (Lap2/Emerin/Man1) subfamily of lamin-associated proteins, Lem2 and Man1 (ref. 54). We detected more than fourfold increase in the sumoylation of Lem2 at K121, a lysine situated in the nucleoplasm, in the *ufd1ΔCt*^*213–342*^ background. Additional five sumoylation sites, clustered near the amino-terminal chromatin-binding Helix–Extension–Helix domain of Lem2, were detected exclusively in the *ufd1ΔCt*^*213–342*^ strain (Fig. 6b and Supplementary Data 2). In addition, a total of nine sumoylation sites were identified in Bqt4, also solely in *ufd1ΔCt*^*213–342*^. Telomere anchoring to the nuclear periphery is known to depend on sumoylation in *S. cerevisiae* and *Caenorhabditis elegans* but the targets of sumoylation are not precisely defined^55^,^56^. Our findings suggest undiscovered modes of telomere–NE regulation through sumoylation of inner membrane proteins. Such regulation is likely to also apply to other heterochromatic regions associating with the NE, such as centromeres. For instance, Lem2 is highly enriched at the centromere/spindle pole body region^54^ and we detected several sumoylation sites on the two-centromere NE-anchoring proteins Sad1 (SUN domain protein) and Csi1 (Fig. 6b). Again, Sad1 and Csi1 sumoylated peptides were almost exclusively detected in the *ufd1ΔCt*^*213–342*^ background. Since the Cdc48–Ufd1–Npl4 ATPase-driven translocation of membrane-bound proteins have been well-established at the endoplasmic reticulum, one may speculate on similar roles for Cdc48–Ufd1–Npl4 in the turnover of NE-associated proteins, possibly via Ufd1 SIM−SUMO interactions.

In addition to proteins associated with centromeres or telomeres, multiple chromatin modifiers and transcription factors were targeted by both Slx8 and Ufd1 including Mot1, a Swi2/Snf2 family ATPase that displaces the TATA-box-binding protein Tbp1 from promoters. Mot1 is an established STUbL substrate in *S. cerevisiae*^12^. We observed increased sumoylation of *S. pombe* Mot1 at K101 and K105 in *slx8-1* and *ufd1ΔCt*^*213–342*^ mutants (Fig. 5a), in a domain of the protein corresponding to the region sumoylated in *S. cerevisiae*. This implicates Cdc48–Ufd1–Npl4 in the processing of sumoylated Mot1. We also detected sumoylation of Tbp1 at K209, within the DNA-binding domain (Supplementary Fig. 5). Other global controllers of transcription and chromatin structure co-regulated by Slx8 and Ufd1 included the SWI/SNF complex subunit Snf59—the histone H3K9 methyltransferase Clr4, the co-repressor Tup12 and the iron-responsive transcription factor Fep1 known to associate with Tup12.

The accumulation of other classes of SUMO conjugates was specific to the *slx8-1* mutant. This was the case for the DNA-directed RNA polymerase I and III complex subunits, for the Cnd2, Cut3 and Cut14 condensin proteins, for the Psm1 and Psm3 core cohesion proteins and for the nucleolar protein Dnt1. Strikingly, Dnt1 showed 1.5- to 7-fold increased sumoylation on 7 different lysines in *slx8-1*. The *S. cerevisiae* Dnt1 homologue, Tof2, is an established SUMO target. Tof2 is part of a network of proteins that tether the ribosomal DNA (rDNA) to the inner nuclear membrane and sumoylation may modulate these interactions^57^. Proper localization of the rDNA at the nuclear periphery is required for retaining rDNA structure and silencing. Consequently, loss of sumoylation leads to decreased rDNA silencing^3^, and an inability to form SUMO chains has been linked to gross defects in rDNA compaction/organization^58^. Moreover, rDNA silencing is also alleviated in STUbL-deficient budding yeast cells^59^, suggesting that a pathway involving both polysumoylation and STUbL activity is required for normal rDNA function. The identification of Dnt1 as a highly upregulated SUMO target in *slx8-1* cells suggests that Dnt1 may constitute a substrate for such a pathway.

Increased sumoylation of the Psm1 and Psm3 core cohesion proteins in *slx8-1* is particularly interesting in light of a recent study conducted in *S. cerevisiae*. Here the other core cohesion subunit Mcd1 (Rad21 homologue) was shown to be targeted for STUbL-mediated degradation in the absence of the cohesin-associated factor Pds5, leading to premature sister chromatid separation^15^. By promoting cohesin proteolysis, STUbLs may allow efficient chromosome segregation during anaphase in wild-type cells^15^ and/or they might regulate cohesion functions outside mitosis such as during DNA repair where the sumoylation of cohesins plays important, yet incompletely defined, roles^60^.

It may be expected that sumoylated proteins normally targeted for degradation by STUbL-catalysed ubiquitylation would accumulate in the *slx8-1* and *ufd1ΔCt*^*213–342*^ backgrounds. However, we found changes in protein sumoylation generally not to be correlated with a change in protein abundance. This lack of correlation, as determined for at least a subset of *slx8-1*- and/or *ufd1ΔCt*^*213–342*^-regulated SUMO substrates, is in line with the notion that only a minor fraction of a protein is typically modified by SUMO at any given time^28^,^61^. Consequently, the total pool of a protein likely to be subjected to STUbL/Ufd1-mediated regulation is proportionally low and any change in total concentration of the target protein may thus go undetected. It is also possible that ubiquitin is added by STUbLs not to degrade sumoylated proteins (that is, K63 rather than K48-linked chains), but rather to allow their extraction from large complexes.

In summary, our SILAC experiments identified a number of proteins whose sumoylation is increased in the *slx8-1* and/or *ufd1ΔCt*^*213–342*^ backgrounds, of which only some were discussed above. We cannot exclude that some conjugates might accumulate through indirect effects of the *slx8-1* or *ufd1ΔCt*^*213–342*^ mutations, for instance, effects on the cell cycle might result in the enrichment of certain cell-cycle-regulated SUMO conjugates. Conversely, our data set might not include particular SUMO-modified peptides, whose chemical composition precludes identification using standard MS-based bottom-up proteomics approaches. This might be the case for a predicted sumoylated peptide^62^,^63^ that was not identified in this study. We trust that the proteins identified include *bona fide* STUbL substrates, based on the finding of already known STUbL targets in the list, and of proteins localizing to subcellular structures or protein complexes previously shown to be affected by STUbL activity. Comparatively few sites displayed reduced sumoylation in either or both mutants. Reductions were limited in both amplitude and number of sites affected, with the remarkable exception of Hmo1 and a predicted ZF-GATA-type transcription factor (SPCC1393.08), which together account for nearly all sites of reduced sumoylation in both the *slx8-1* and *ufd1ΔCt*^*213–342*^ mutants (Fig. 5). Common tendencies in sumoylation dynamics between the *slx8-1* and *ufd1ΔCt*^*213–342*^ mutant strains lend support to recent genetics observations of a functional relationship between STUbLs and the Cdc48–Ufd1–Npl4 segregase, and should motivate the further characterization of the specific substrates through which STUbL and Cdc48–Ufd1–Npl4 act either in concert or individually.

## Methods

### Strain constructions

The strains used in this study are listed in Supplementary Table 1. The endogenous *S. pombe* SUMO gene, *pmt3*, was engineered to encode a SUMO variant with an N-terminal His_6_-tag and a single amino-acid substitution changing the leucine at position 109, immediately upstream the C-terminal diGly sequence, into a lysine (*His*_*6*_*SUMO*^*L109K*^ allele). This was performed using a ‘loop-in-loop-out' strategy^64^, where a plasmid containing *His*_*6*_*SUMO*^*L109K*^, *pmt3*^*+*^ 5′ and 3′ flanking regions and a *ura4*^*+*^ gene (pJBK142) was integrated into the *pmt3*-flanking region by homologous recombination in a *ura-D18* strain. ‘Loop-outs' where *His*_*6*_*SUMO*^*L109K*^ remained as the single *pmt3* genomic copy were subsequently selected by 5′-fluoroorotic acid resistance and they were verified by PCR amplification of the locus and sequencing. To produce the pJBK142 ‘loop-in' plasmid, the *S. pombe* SUMO (*pmt3*) open reading frame and its flanking regions were amplified in two separate PCR reactions: The open reading frame and its 5′ region were amplified with the forward primer GTO-492 (5′- GCTCCACAATTCTCACAAGCACCC -3′) and reverse primer GTO-548 (5′- CGATGGATCCCTAAAGGCATAGATGGGTGCAACCACCTTTCTGTTCTAAG -3′), changing the codon for leucin 109 (TTA) to a lysine codon (TTT), as underlined. The *pmt3* downstream region was amplified with GTO-549 (5′- GCATGGATCCAGTACAAGTATTTTTAAGCTGTTTC -3′) and GTO-550 (5′- GCATCCATGGCGCTCAGTCAAGGTATGCTTCCATAAGCAG -3′). Prior to chromosomal integration, the plasmid was digested with *SphI*. After *His*_*6*_*SUMO*^*L109K*^ allele replacements had been identified, a *ura4*^*+*^ gene was inserted at a tightly linked location 5′ of *pmt3* to follow the allele through subsequent crosses. *His*_*6*_*SUMO*^*L109K*^ *int::pJBK111(ura4*^*+*^) was crossed into *slx8-1* and *ufd1ΔCt*^*213–342*^ mutant backgrounds and combined with the *lys3–27* allele using standard genetic crosses.

### Anti-SUMO western blots

The indicated strains were grown in yeast-extract medium at 30 °C and harvested at an *A*_600_ ∼0.5. Cells were lysed with the glass bead method in a denaturing buffer containing 8 M urea; 100 mM NaHPO_4_ pH 7.5; 50 mM Tris-HCl pH 7.5; 150 mM NaCl and 20 mM N-ethylmaleimide using a FastPrep instrument (MP Biomedicals). Equal amounts of protein lysates were separated by SDS–polyacrylamide gel electrophoresis on 4–20% gradient gels (Lonza), transferred to a nitrocellulose/MCE membrane (Advantec) and immunoblotted using an anti-SUMO rabbit antibody (kindly provided by J. Seeler; diluted 1:2,000). Following incubation with a horseradish peroxidase-conjugated swine anti-rabbit IgG secondary antibody (Dako PO217, diluted 1:50,000), sumoylated species were detected with an ECL plus kit (GE Healthcare). Western blots detecting tubulin were performed in parallel with an anti-tubulin antibody (Sigma 00020911; diluted 1:25,000).

### Cultures and lysis for label-free total proteome analysis

Strains JK424, JK354 and JK408 were grown in triplicates in Edinburgh minimal medium (EMM2) supplemented with 30 mg l^−1^ lysine at 30 °C, harvested at an OD(*A*_600_)∼1 and washed in ice-cold PBS before freezing in liquid nitrogen. Cells were lysed with NaOH/β-mercaptoethanol, as described below for SILAC experiments in section ‘Protein extraction and nickel affinity purification', and resuspended in 6 M guanidinium-HCl; 100 mM NaHPO_4_, pH 8.0; 10 mM Tris-HCl, pH 8.0; 20 mM imidazole; 5 mM β-mercaptoethanol.

### Culture conditions for non-SILAC experiment

The *ufd1ΔCt*^*213–342*^ *His*_*6*_*SUMO*^*L109K*^ strain JK414 was grown in EMM2 supplemented with 30 mg l^−1^ lysine at 30 °C. Approximately 2.5 l of culture were harvested from exponentially growing cells (*A*_600_∼0.9) and washed in ice-cold PBS before freezing in liquid nitrogen.

### Culture conditions for SILAC

Strains were grown in EMM2 supplemented with 30 mg l^−1^ of ‘light' (^12^C_6_^14^N_2_, Lys0) or ‘heavy' L-lysine (^13^C_6_^15^N_2_, Lys8) (Cambridge Isotope Laboratories). The wild-type (JK408) and *ufd1ΔCt*^*213–342*^ (JK414) strains were each grown in light medium and individually compared with the *slx8-1* (JK418) strain grown in parallel in heavy medium. The experiments were performed in triplicates starting from independent biological isolates. To ensure full incorporation of heavy lysine, the *slx8-1* strain was propagated in SILAC medium for at least 25 generations after which the incorporation efficiency was determined to be more than 98%. The cultures were propagated at 30 °C, except for the last 12 h where they were propagated at 33 °C to induce the *slx8-1* temperature-sensitive phenotype at a semi-permissive temperature^5^. Approximately 2 l of culture (*A*_600_∼1) were harvested per strain per experiment. Cell pellets were washed in ice-cold PBS and frozen in liquid nitrogen.

### Protein extraction and nickel affinity purification

Protein extraction and Ni^2+^ purification of His_6_SUMO^L109K^ conjugates from *ufd1ΔCt*^*213–342*^ unlabelled cultures and from SILAC cultures were performed using similar protocols. The SILAC experiments, comparing *slx8-1* with wild type and *slx8-1* with *ufd1ΔCt*^*213–342*^, were processed in parallel. Heavy and light labelled cells were mixed in a 1:1 ratio based on the weight of cell pellets prior to lysis. Mixed pellets were resuspended in 50 ml ice-cold H_2_O and lysed by adding an equal volume of NaOH and β-mercaptoethanol to a final concentration of 1.85 M and 1.85%, respectively (∼12 ml lysis buffer per 1 g of cell pellet). After incubation on ice for 30 min, proteins were precipitated with 25% trichloroacetic acid, washed with ice-cold acetone and resuspended in 60 ml of binding buffer (6 M guanidinium-HCl; 100 mM NaHPO_4_, pH 8.0; 10 mM Tris-HCl, pH 8.0; 20 mM imidazole; 5 mM β-mercaptoethanol). Proteins were allowed to go into solution in binding buffer on a roller mixer for up to 1 h before lysates were finally clarified by high-speed ultracentrifugation (30,000*g* for 20 min). In all cases, the total protein yield was ∼1.5 g as determined by ultraviolet–visible spectrophotometry (*A*_280_). Approximately 100 μg of cell lysates were put aside for complete proteome analysis while the remaining material was mixed with 5 ml of Ni^2+^-NTA agarose beads (Qiagen) pre-equilibrated in binding buffer, and the slurry was incubated at 4 °C overnight. Beads were collected and washed on gravity columns with 50 ml of each buffer in the following order: binding buffer, wash buffer pH 8.0 (8 M urea; 100 mM NaHPO_4_, pH 8.0; 10 mM Tris-HCl, pH 8.0; 20 mM imidazole; 5 mM β-mercaptoethanol), wash buffer pH 6.3 (8 M urea; 100 mM NaHPO_4_, pH 6.3; 10 mM Tris-HCl, pH 6.3; 20 mM imidazole; 5 mM β-mercaptoethanol) and once more with wash buffer pH 8.0. Proteins were eluted in three sequential steps with 5 ml of wash buffer pH 8.0 containing 200 mM imidazole and the three elution fractions of each biological sample were combined for further processing.

### Filter-aided sample preparation and protein digestion

His_6_SUMO^L109K^-conjugated proteins (∼300 μg as determined by ultraviolet–visible spectrophotometry) were digested on 30-kDa cut-off filter units (Sartorius, Vivacon 500) essentially as described in the study by Tammsalu *et al*.^65^ Enriched protein samples were concentrated onto three filters. Each filter was washed twice with 200 μl UA buffer (8 M urea; 100 mM Tris-HCl pH7.5) and treated with 50 mM chloroacetamide in UA buffer for 20 min in the dark. After two additional washes with UA buffer, the samples were equilibrated three times with 200 μl of IP buffer (50 mM MOPS-NaOH pH 7.2; 10 mM Na_2_HPO_4_; 50 mM NaCl). Proteins were digested with Lys-C (Wako) in 50 μl of IP buffer for 16 h at 37 °C at a 1:50 enzyme-to-protein ratio. The digested peptides were collected by centrifugation and filter units were washed with 50 μl of IP buffer to increase the peptide yield. To recover high-molecular weight peptides retained on filters, another digestion step was performed with Glu-C (1:100 enzyme-to-protein ratio) in 50 μl IP buffer for 16 h at 20 °C. Peptides collected after Lys-C or Lys-C and Glu-C digestions were kept separately.

For complete proteome analysis, 100 μg of protein in binding buffer was diluted 10-fold into 8 M urea, 100 mM Tris-HCl, pH 8.0 and treated with 50 mM chloroacetamide in the dark at room temperature for 1.5 h. The samples were then diluted fivefold with 50 mM ammonium bicarbonate to reduce the urea concentration and digested in solution with Lys-C (1:50 enzyme-to-protein ratio) for 16 h at 20 °C. Resulting peptides were fractionated into six fractions based on the pH of the solution (pH 11.0, 8.0, 6.0, 5.0, 4.0 and 3.0) used to elute the peptides from pipette tip-based anion exchanger (3 M Empore Anion Exchange-SR)^66^.

Label-free quantification (LFQ) was performed using protein from *S. pombe* cells expressing wild-type, His_6_SUMO or His_6_SUMO^L109K^ as their sole copy of SUMO. Approximately 20 μg of protein from an *S. pombe* cell lysate in binding buffer was diluted tenfold into 8 M urea; 100 mM Tris-HCl, pH 8.0 and treated with 50 mM choloroacetamide in the dark for 1.5 h at room temperature. Urea concentration in the sample was then diluted fourfold using 50 mM ammonium bicarbonate. Proteins were digested in solution for 4 h at room temperature using Lys-C (1:50 enzyme-to-protein ratio) before the samples were diluted by another twofold with 50 mM ammonium bicarbonate. Resulting peptides were digested with trypsin (1:50 enzyme-to-protein ratio) overnight at room temperature before the proteases were inactivated by the addition of trifluoroacetic acid (TFA) to a final concentration of 1%.

### DiGly–Lys-specific IP

An anti-KɛGG antibody coupled to protein A beads (PTMScan, Cell Signaling Technology) was used for IP of diGly-modified peptides. Prior to use, coupled beads were washed twice with 20 bead volumes of conjugation buffer (20 mM NaHPO_4_, pH 9.0; 150 mM NaCl) and crosslinked in 25 bead volumes of 5 mM Bis(sulfosuccinimidyl)suberate for 30 min at room temperature while rotating. The reaction was quenched by adding 1 M Tris-HCl, pH 7.5 to a final concentration of 50 mM and rotating the samples for 15 min at room temperature. Crosslinked beads were washed three times with 20 bead volumes of cold IP buffer (50 mM MOPS-NaOH, pH 7.2; 10 mM Na_2_HPO_4_; 50 mM NaCl) and resuspended in IP buffer (50% slurry). About 3 μl of beads crosslinked to 19 μg of anti-KɛGG antibody were added to Lys-C- or Lys-C- and Glu-C-digested peptide mixtures in IP buffer and incubated at 4 °C overnight. The beads were washed twice with 500 μl of ice-cold PBS and peptides were eluted in three sequential steps with 50 μl of 0.15% TFA. Three elution fractions of each biological sample were combined.

### MS analysis

All peptide samples were desalted and concentrated with home-made reverse-phase C18 (Empore) Stop and Go extraction Tips^67^, resuspended in 0.1% TFA and analysed by LC-MS/MS. An EASY-nLC 1,000 liquid chromatography system (Thermo Scientific) coupled to a Q Exactive mass spectrometer (Thermo Scientific) through an EASY-Spray ion source (Thermo Scientific) was used and purified peptides were separated on an EASY-Spray column (75 μm × 500 mm). Samples containing SUMO remnant-modified peptides, Lys-C peptides corresponding to complete proteome or tryptic peptides for the LFQ were analysed with a 90, 240 or 150 min linear gradient of acetonitrile in 0.1% formic acid, respectively, at a flow rate of 250 ml per min, and the majority of peptides eluted during a 72-, 220-, 130-min acetonitrile window from 5 to 40% or from 5 to 50%. MS and MS/MS spectra of the eluting peptides were acquired online by Q Exactive mass spectrometer operated in a data-dependent mode. Precursor ion full-scan MS spectra were acquired at a target value of 1,000,000 ions (maximum injection time 20 ms) over a scan range of 300 to 1,600 Th or 300 to 1,800 Th with a resolution of 70,000 at *m/z* 400. For samples following diGly–Lys-specific enrichment, up to one data-dependent higher energy collisional dissociation (HCD) MS/MS was acquired at a target value of 500,000 ions (maximum injection time 1,000 ms) with a resolution 35,000 at *m/z* 400. Up to 10 HCD MS/MS spectra were acquired for complete proteome and LFQ analyses at a target value of 500,000 ions (maximum injection time 60 ms) with a resolution 17,500 at *m/z* 400. Normalized collision energy was set to 30%, singly or highly (>8) charged ions or ions with unassigned charge states were rejected, *m/z* values of acquired ions were added to the exclusion list for 40 s and peptide match option was set to preferred.

### Data analysis

Raw mass spectrometric data files were processed with MaxQuant software (version 1.3.0.5)^36^ and searched against UniprotKB *S. pombe* reference protein database containing canonical and isoform sequences (downloaded on 14 December 2013). UniprotKB gene names were used in Tables except for a few names that were updated with Pombase gene names. Protease specificity was set for Lys-C or Lys-C and Glu-C permitting up to three or five missed cleavages, respectively. Carbamidomethylation of Cys residues was added as a fixed modification and oxidation of Met, acetylation of protein N-termini and diGly adduction to internal Lys in the peptide sequence were set as variable modifications. In addition, phosphorylation of Ser, Thr and Tyr or acetylation of Lys were added as variable modifications and the minimum peptide length was set to seven amino acids. For SILAC samples, multiplicity was set to 2, and Lys8 was selected as a heavy labelled counterpart. LFQ files were analysed using MaxQuant software version 1.5.2.8. Multiplicity was set to 1, trypsin was selected as an enzyme and up to two missed cleavages were permitted. LFQ^68^ and match between runs options were both enabled with a minimum LFQ ratio count of 2, match time window of 1 min and alignment time window of 20 min. All data sets were filtered by posterior error probability to achieve a false discovery rate of 1% at both, protein and peptide level.

Complete proteome results (Supplementary Data 4) report only proteins that were quantified in at least one biological replicate. Potential contaminants and proteins identified based on reverse decoy database search were excluded. For each quantified protein, the normalized protein ratio of individual biological replicates was averaged and expressed as a log_2_. The number of ratio occurrences and the variance of these ratios between the individual biological replicates of each experimental condition are reported in Supplementary Data 4. Mean normalized ratio of JK414/JK408 was derived from the mean ratios of JK418/JK408 and JK418/JK414, and was expressed as a log_2_. The list of proteins quantified in the LF experiment (Supplementary Data 1) includes proteins that were quantified in at least two biological replicates of one experimental condition, omitting potential contaminants and proteins identified based on reverse decoy database search. LFQ values of the individual biological replicates were averaged and the ratio of the values of His_6_SUMO/SUMO and His_6_SUMO^L109K^/SUMO were expressed as a log_2_. Each protein in the 2D scatter plot was coloured using a gradient from green to red according to the density estimation values calculated using Perseus software package (300 points per dimension; P(*x*,*y*) density function; version 1.5.2.6; downloaded from http://141.61.102.17/perseus_doku/doku.php?id=start). Perseus software was also used to calculate multiple-sample analysis of variance test −log *P* values (false discovery rate=0.01; *s*_0_=0.14). The list of modification sites (Supplementary Data 2) contains peptides encompassing a GlyGly-modified lysine identified from any of the immunoaffinity purification experiments. Potential contaminants, peptides identified using reverse decoy database search and peptides containing modification sites with a localization probability <75% were excluded. Peptide precursor mass error was checked to be <2 p.p.m. after mass recalibration or 4 p.p.m. in case the recalibration was unsuccessful. GlyGly-modified peptides quantified in a minimum of two biological replicates in at least one experimental condition were reported in the list of quantified modification sites (Supplementary Data 3). The non-normalized heavy-to-light ratio of every peptide reported in the MaxQuant output file was manually normalized according to the median normalization factor obtained from the MaxQuant output file of the corresponding complete proteome experiment that contained non-normalized and normalized protein ratios (‘experiment 1' median normalization factor=‘experiment 1' complete proteome median normalized heavy-to-light ratio/‘experiment 1' complete proteome median non-normalized heavy-to-light ratio). For each quantified diGly-modified peptide, a median normalized ratio of individual biological replicates was reported and expressed as a log_2_. The number of ratio occurrences and the variance of these ratios between the individual biological replicates of each experimental condition were added to the Supplementary Data 3. Median normalized ratio of JK414/JK408 was derived from the median ratios of JK418/JK408 and JK418/JK414, and was expressed as a log_2_. The table of peptides with additional PTMs (Supplementary Data 6) includes entries where the localization probability of the additional modification is >75%, the mass error of the precursor ion is <2 p.p.m. and the peptide has not been detected using reverse decoy database search. MS/MS spectra of peptides with additional modifications and their corresponding sequences modified by diGly only have been manually validated on the basis of good coverage of y- and b-ion series and extensive identification of intensive fragment ion peaks.

### Analysis of His_6_SUMO^L109K^-modified sites

Sequence motifs were visualized using pLogo^69^. Sequence windows of 13 amino acids centred on all SUMO-modified lysines identified were used as input. Protein N- or C-terminal sequences shorter than the 13-amino-acid window were omitted from the output. UniprotKB *S. pombe* reference protein database (downloaded on 14 December 2013) containing 145,132 lysines was used as a background data set and residues were scaled relative to their Bonferroni-corrected statistical significance.

### Mapping of sumoylation sites onto existing protein structures

The ensemble of *S. pombe* proteins for which sumoylation sites were identified in this study (Supplementary Data 2) were used in a Blast search at NCBI against the Protein Data Bank. PDB files were retrieved for proteins with high identity with their *S. pombe* homologues. Lysines that are sumoylated in *S. pombe* were mapped onto structures with the PyMOL Molecular Graphics System, Version 1.3 Schrödinger, LLC. The data are displayed in Fig. 3c,d, Supplementary Figs 4 and 5 and in Supplementary Data 7.

### PSIPRED analysis

PSIPRED (version 3.5 for Linux; derived from the study by Jones^70^) and the Uniref90 database (release 2014_10) were used to predict the secondary structure context (that is, helix, sheet, coil) of all identified sumoylated lysines and of all lysines in the same proteins (Supplementary Data 8 and Fig. 3e). The secondary structure context at positions +3 and −3 was also determined, using a custom script.

## Additional information

**Accession codes.** The raw mass spectrometry data and the associated tables have been deposited to the ProteomeXchange Consortium (http://proteomecentral.proteomexchange.org) via the PRIDE partner repository with the data set identifier PXD002972.

**How to cite this article:** Køhler, J. B. *et al*. Targeting of SUMO substrates to a Cdc48–Ufd1–Npl4 segregase and STUbL pathway in fission yeast. *Nat. Commun.* 6:8827 doi: 10.1038/ncomms9827 (2015).

## Supplementary Material

Supplementary Information Supplementary Figures 1-7, Supplementary Table 1 and Supplementary References

Supplementary Data 1 Comparison of proteomes in cells expressing wild-type SUMO, His_6_SUMO, or His_6_SUMO^L109K^.

Supplementary Data 2 Sumoylated residues identified in all experiments combined. Sites identified in the *ufd1δ^Ct213-342^* unlabeled and in the SILAC experiments are indicated in separate columns by a "+". "Number of GlyGly sites on peptide" refers to whether a peptide was identified with a single, a double or a triple diGly-Lys modification. The MaxQuant calculated score values (Andromeda) are displayed for individual sites_8_.

Supplementary Data 3 Sumoylated residues quantified in the SILAC experiments. Some sites were quantified as part of doubly or triply-modified peptides as indicated.

Supplementary Data 4 Proteins quantified in crude SILAC lysates.

Supplementary Data 5 Absolute abundance of proteins detected in this study. Values were extracted from Table S8 in Marguerat et al. ^(2012)1^.

Supplementary Data 6 Sumoylated peptides co-modified by phospho or acetyl groups.

Supplementary Data 7 Analysis of sumoylation sites mapped onto protein structures. PDB numbers are indicated, as well as the % identity shared between each *S. pombe* protein and its PDB match. The position and amino acid context of lysines are also indicated for both the identified *S. pombe* sumoylation sites and their homologous lysine in the crystal or NMR structures. The structures were used to determine whether the lysines are in coils, close to the ends of alpha helices or beta sheets (i.e. within three amino acids to the end of an alpha helix or a beta sheet), or more internal to alpha helices or beta sheets. The numbers are presented in a bar graph in Fig. 3d. Cartoons of the structures with highlighted sumoylation sites are presented in Fig. 3c and Supplementary Fig. 4 and 5.

Supplementary Data 8 PSIPRED analysis of sumoylation sites. Supplementary Table 8 was used to prepare Fig. 3e.

Supplementary Data 9 Comparison of *S. pombe* and *S. cerevisiae* sumoylated proteomes. The sources were Wohlschlegel et al.^3^; Hannich et al.^4^ Denison et al. ^5^; Panse et al. ^6^; Albuquerque et al. ^7^. The data were generated with YeastMine2 (2015-05-31 version) and used to generate Supplementary Fig. 6.
