# Supplementary material for: Targeting of SUMO substrates to a Cdc48–Ufd1–Npl4 segregase and STUbL pathway in fission yeast
Source: Nat Commun. 2015 Nov 5;6:8827. doi: 10.1038/ncomms9827 (PMC4667616; doi:10.1038/ncomms9827)
Supplement: Supplementary Information — Supplementary Figures 1-7, Supplementary Table 1 and Supplementary References [file ncomms9827-s1.pdf]

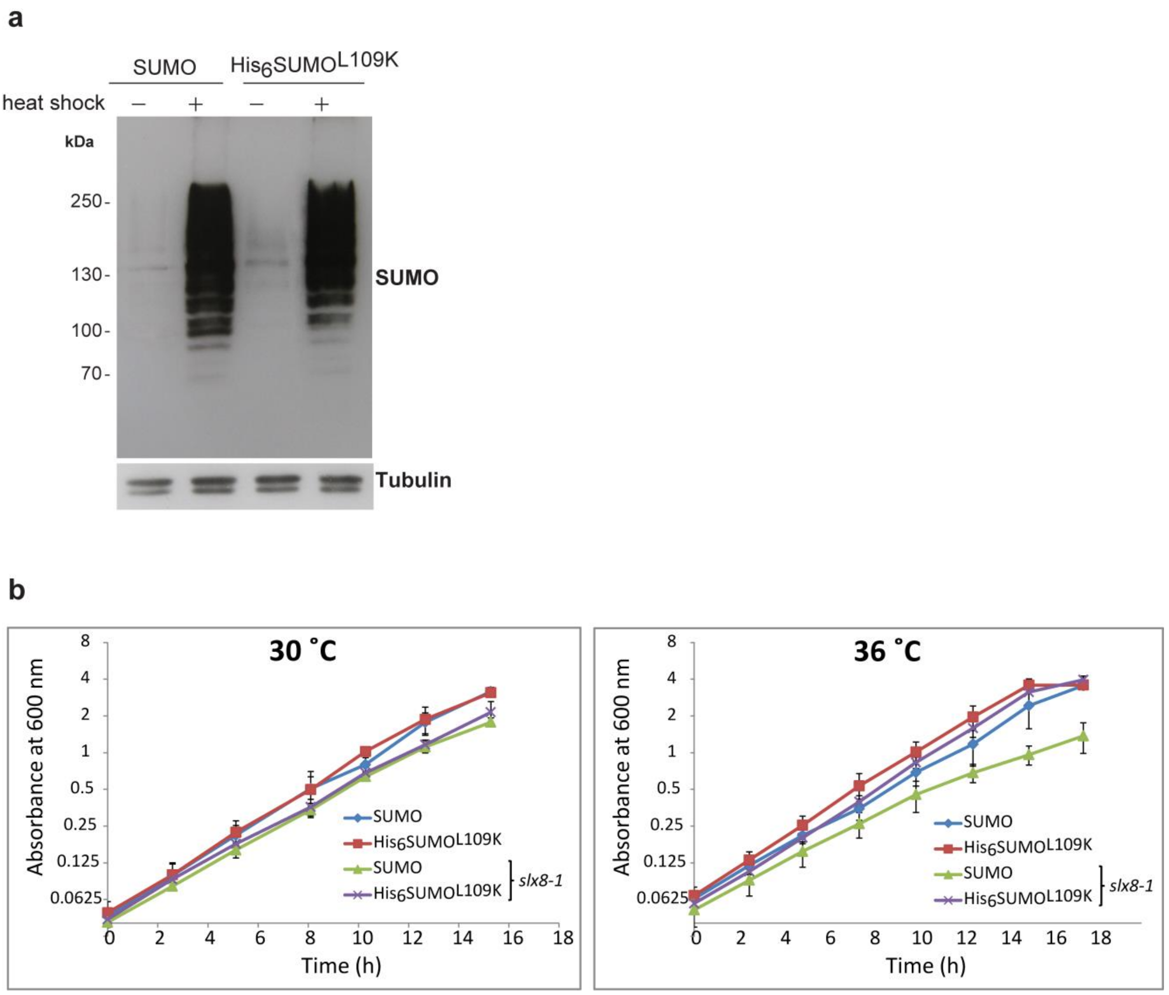

Supplementary Figure 1. Supplementary phenotypic analyses of cells expressing His$_6$SUMO^{L109K}. (**a**) Heat shock response. Strains expressing wild-type SUMO or His$_6$SUMO^{L109K} were propagated at 30°C and heat shocked at 42°C for 20 min as indicated. Protein extracts were probed with a SUMO antibody as in Fig. 1. (**b**) Growth at 30°C and 36°C. Wild-type and mutants were propagated at 30°C or 36°C as indicated and OD_{600} measurements were taken to determine growth rates. Standard errors were computed from three independent cultures for each strain.

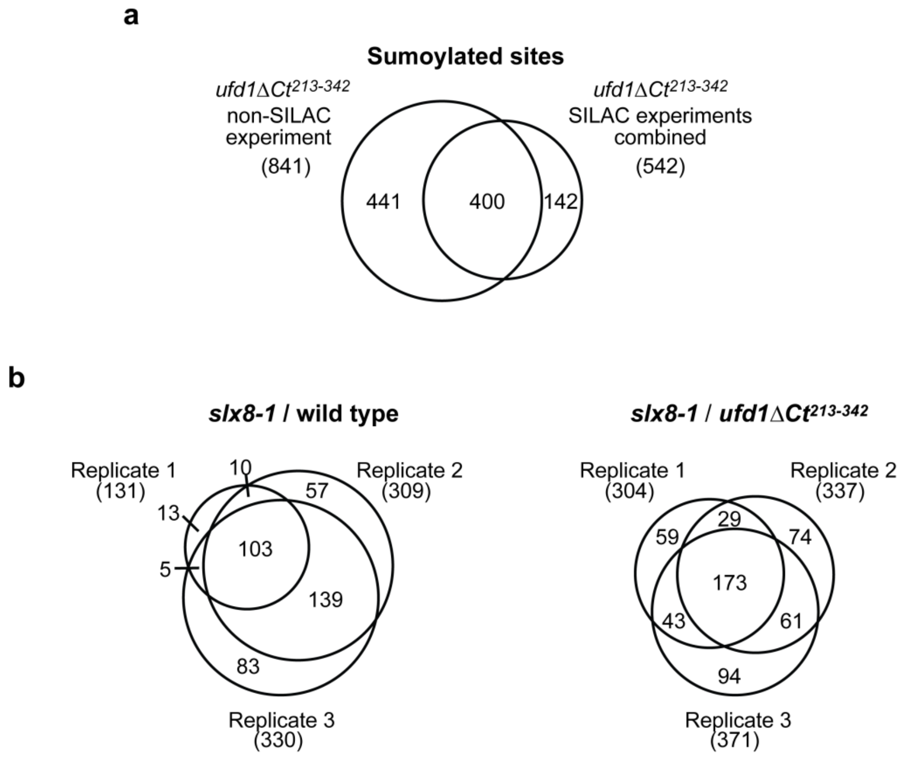

Supplementary Figure 2. Overview of experimental reproducibility. (**a**) Venn diagram comparing sites identified in the unlabeled *ufd1ΔCt*$^{213\text{-}342}$ His_6-$SUMO^{L109K}$ IP with sites identified in the same *ufd1ΔCt*$^{213\text{-}342}$ strain (JK414) in the SILAC biological triplicates combined. (**b**) Venn diagrams comparing sites identified in the biological triplicates processed for each SILAC experiment. The data for Supplementary Fig. 2 can be found in Supplementary Table 2 and 3.

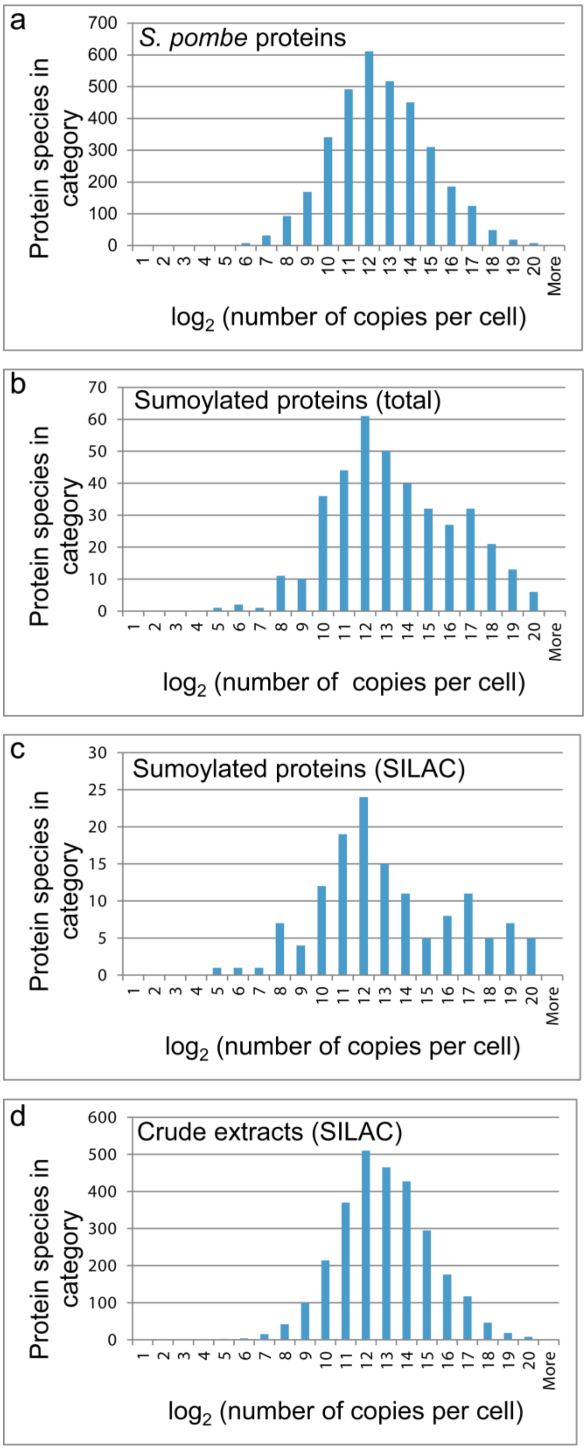

Supplementary Figure 3. Distribution of proteins identified in this study according to absolute abundance. An *S. pombe* large-scale study by Marguerat *et al.* (2012) [1] determining the number of protein molecules per cell was used to generate histograms for (**a**) the proteome of exponentially growing wild-type *S. pombe* (Marguerat *et al.* Table S8); (**b**) the proteins in our Supplementary Table 2; (**c**) the proteins in our Supplementary Table 3; and (**d**) the proteins in our Supplementary Table 4. In all cases, the 'number of copies per cell' refers to the number of protein molecules per cell determined by Marguerat *et al.* for individual *S. pombe* protein species. The analysis shows that our detection is not limited to highly abundant proteins. Values are shown in Supplementary Table 5.

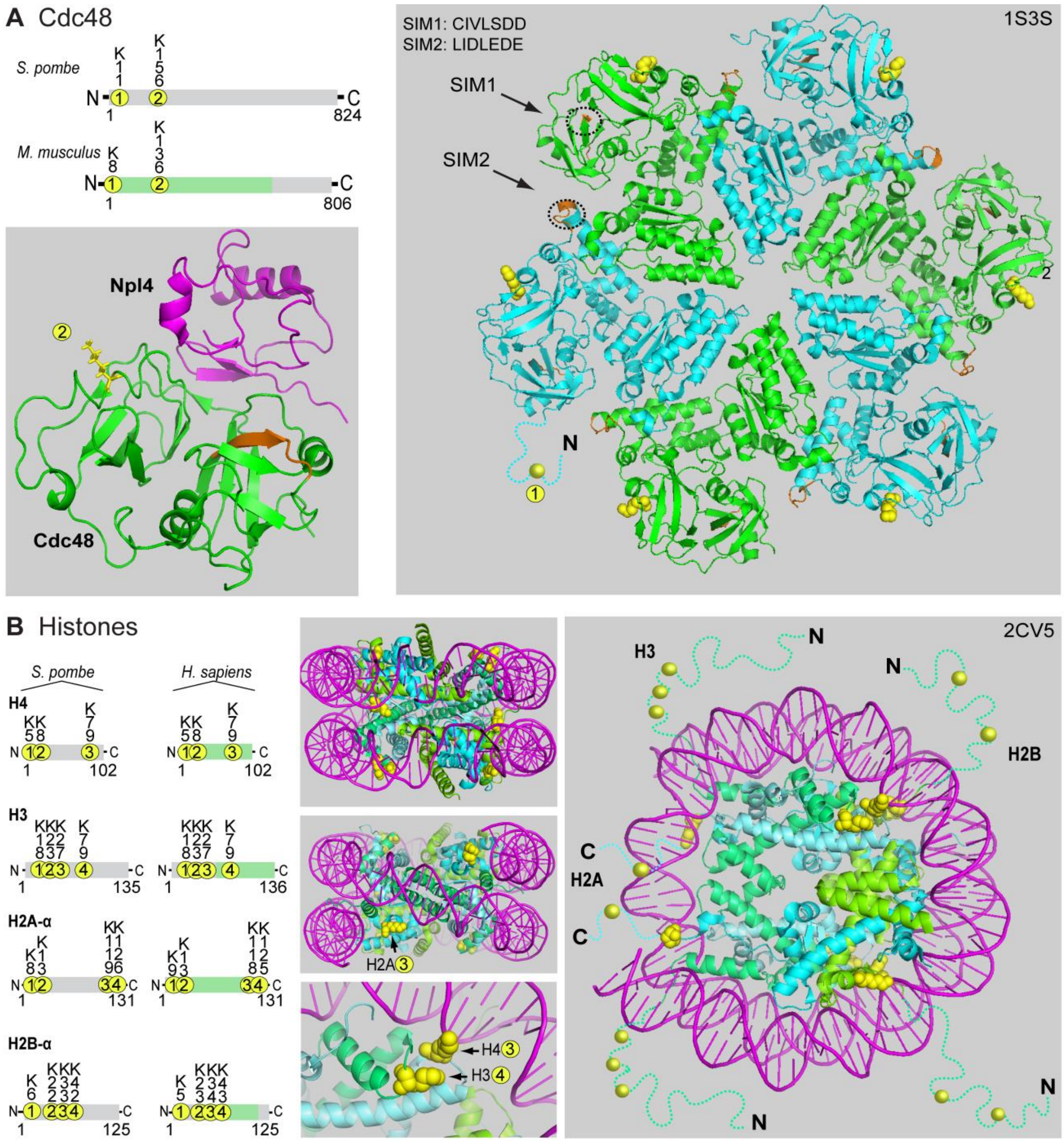

Supplementary Figure 4. Sumoylation sites of Cdc48 and histones. (**a**) Cdc48. The *S. pombe* protein is 70 % identical to mouse Cdc48 (PDB#1S3S). *S. pombe* Cdc48 is sumoylated at K11 and K156 but only K156 is present in the crystal structure. K156 is close to a groove between the two amino-terminal subdomains of Cdc48 where the interactions of Cdc48 with Ufd1/Npl4 and other co-factors take place as shown on the left in a Npl4-Cdc48 co-crystal (PDB#2PJH). Sumoylation at K156 might therefore affect the interactions of Cdc48 with its co-factors. An experimentally characterized SIM (SIM1), and a putative SIM (SIM2) are indicated in 1S3S. Internal SIM-SUMO interactions might take place within Cdc48, perhaps reducing the accessibility of Cdc48 to co-factors, or bridging adjacent Cdc48 subunits. (**b**) Histone octamer. Sumoylation sites detected in histone H2A, H2B, H3, and H4, were mapped on the human nucleosome structure (PDB#2CV5). Sequence identity at the amino acid level is respectively 92% for histone H3, 90% for histone H4, 83% for histone H2A and 70% for histone H2B. All sites are accessible in the presence of DNA, except for H2BK33. The amino acid numbering shown in this Figure for *S. pombe* are for mature histones lacking the initiating methionine; consequently they differ from the numbering in Supplementary Tables by one amino acid. The first 16 amino acids of histone H4 are missing in 2CV5.

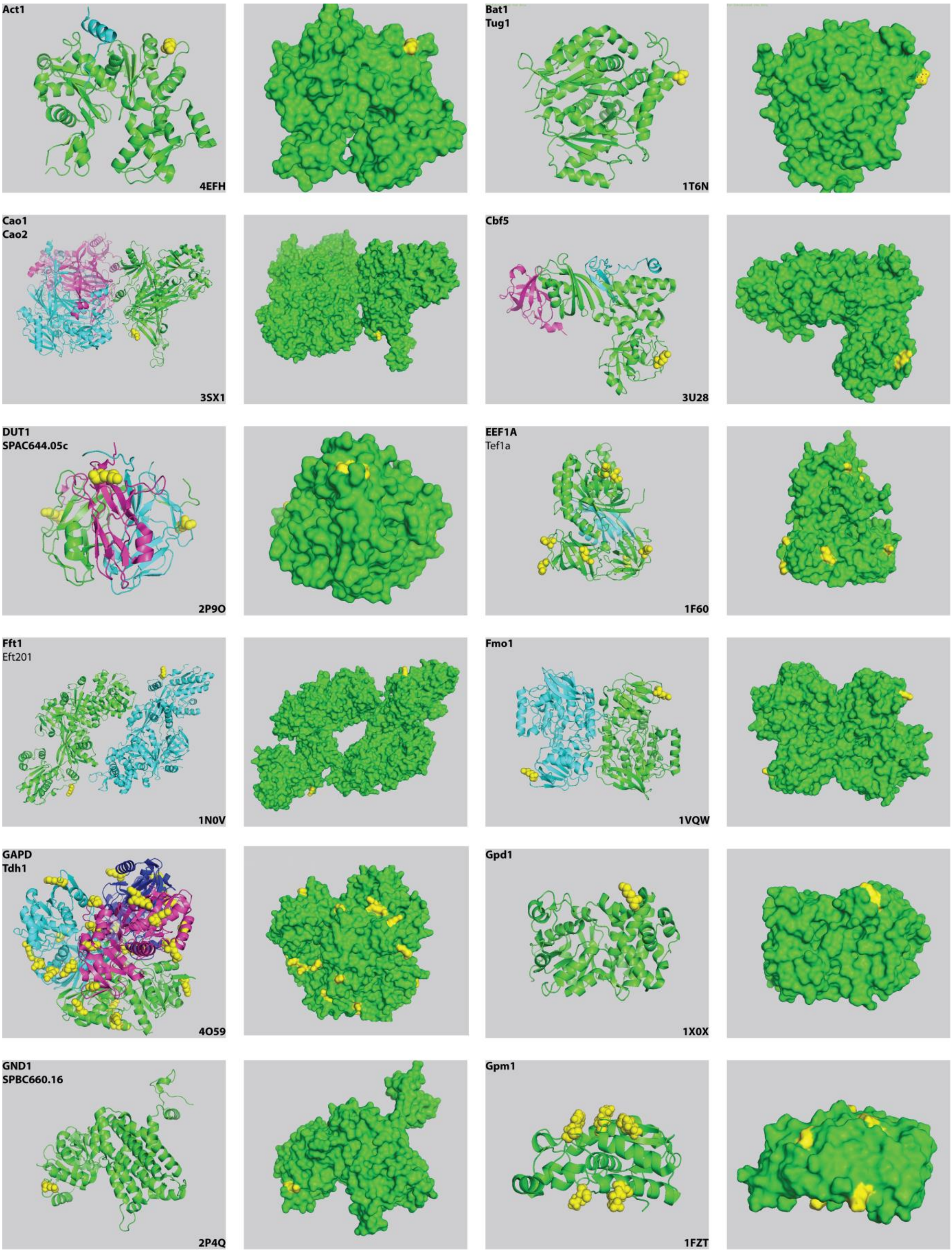

Supplementary Figure 5.

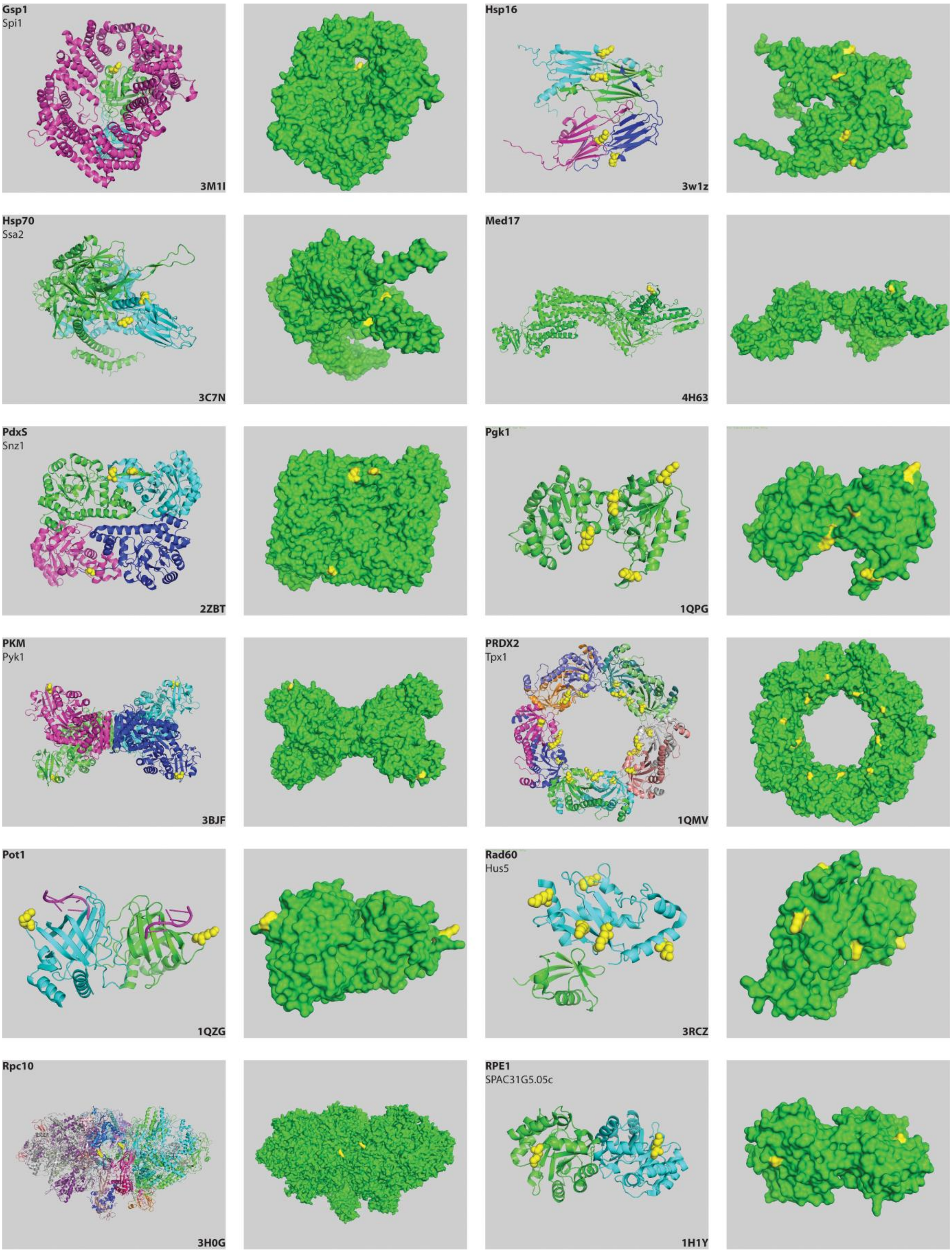

Supplementary Figure 5.

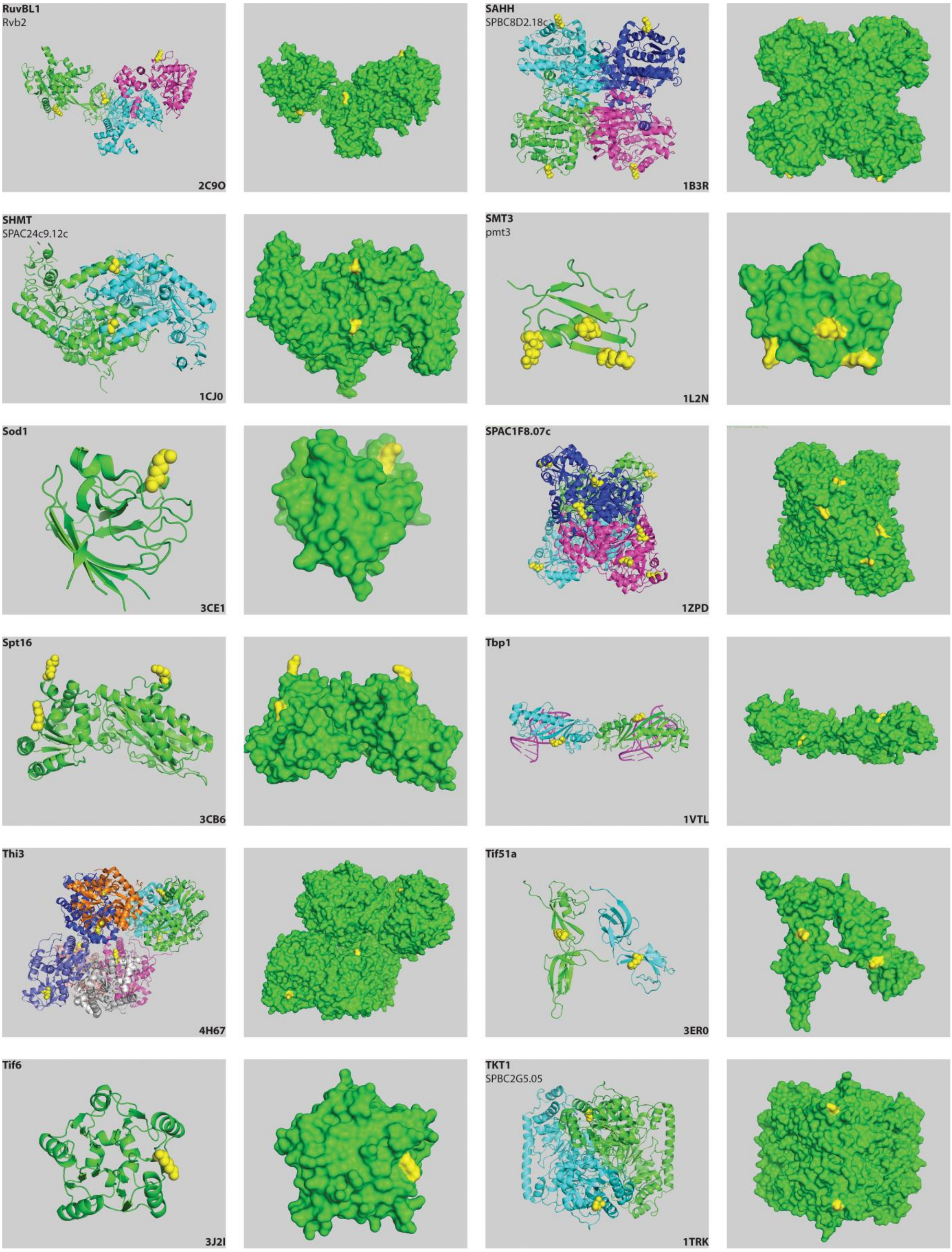

Supplementary Figure 5.

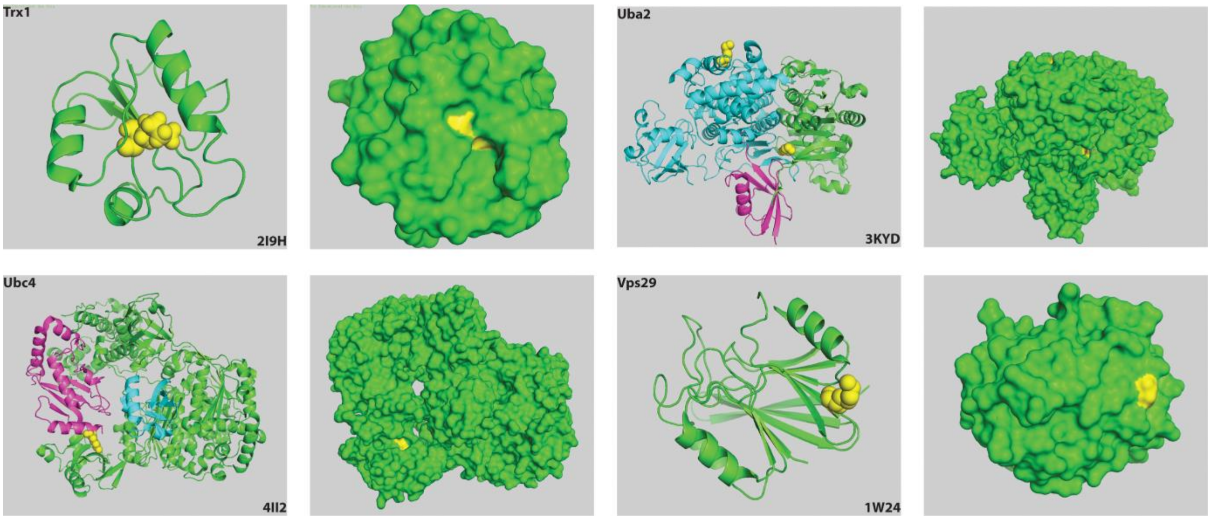

Supplementary Figure 5. Mapping of sumoylation sites onto crystal structures. Sumoylation sites identified in this study for *S. pombe* were mapped onto existing crystal structures. They are highlighted here in yellow. The PDB accession numbers of the structures used are indicated. Sequence alignments and % identity between the *S. pombe* proteins and the crystalized proteins are shown in Supplementary Table 7.

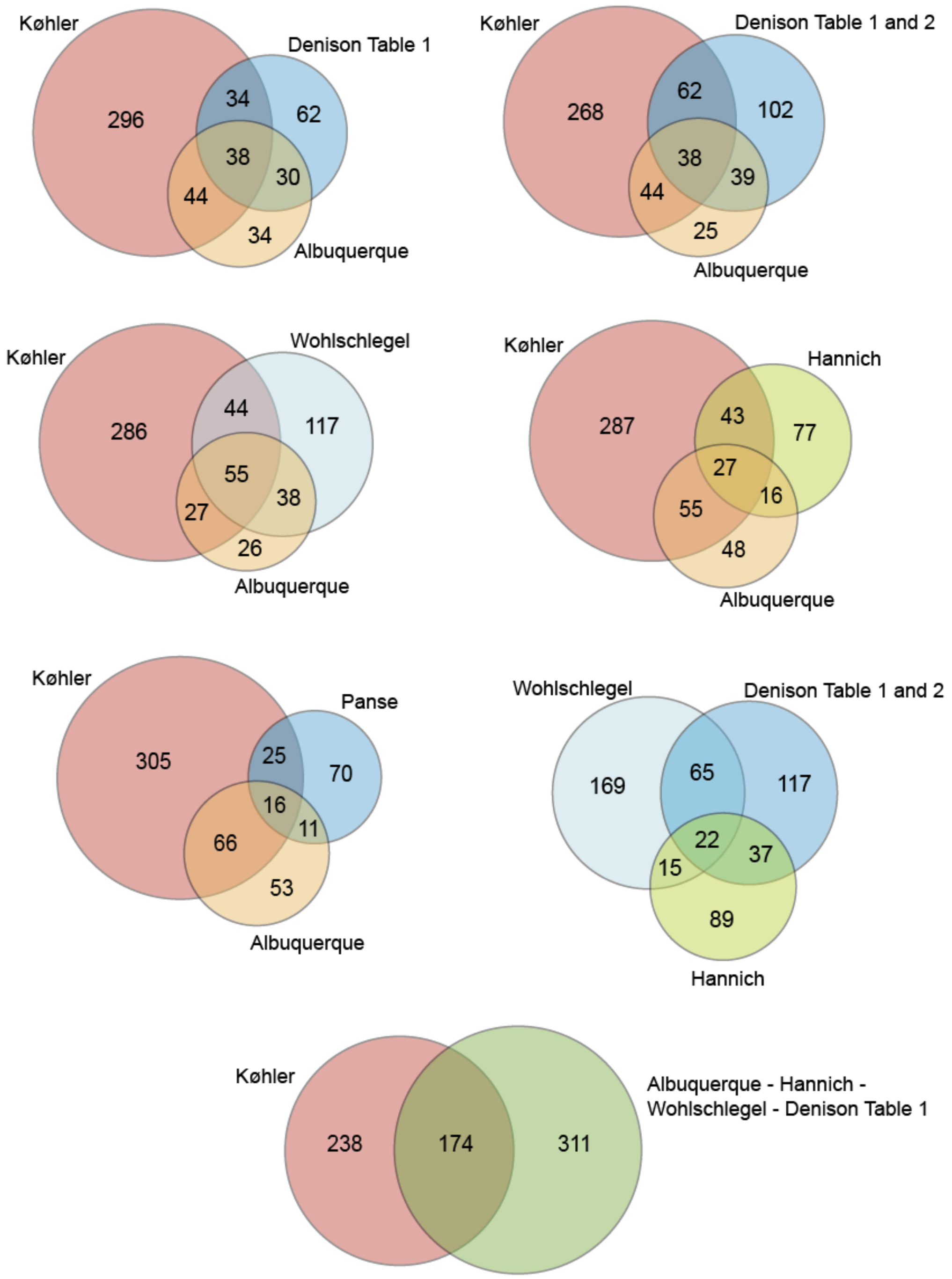

Supplementary Figure 6. Comparison of *S. pombe* and *S. cerevisiae* sumoylated proteomes. The *S. pombe* sumoylated proteins displayed in Supplementary Table S2 that have orthologues in *S. cerevisiae* according to YeastMine[2] (2015-05-31 version) were compared with *S. cerevisiae* proteins with identifiable orthologues in *S. pombe*. The sources were Wohlschlegel *et al.*[3]; Hannich *et al.* [4]; Denison *et al.* [5]; Panse *et al.* [6]; Albuquerque *et al.* [7]. The protein lists used to generate the Venn diagrams are shown in Supplementary Table 9.

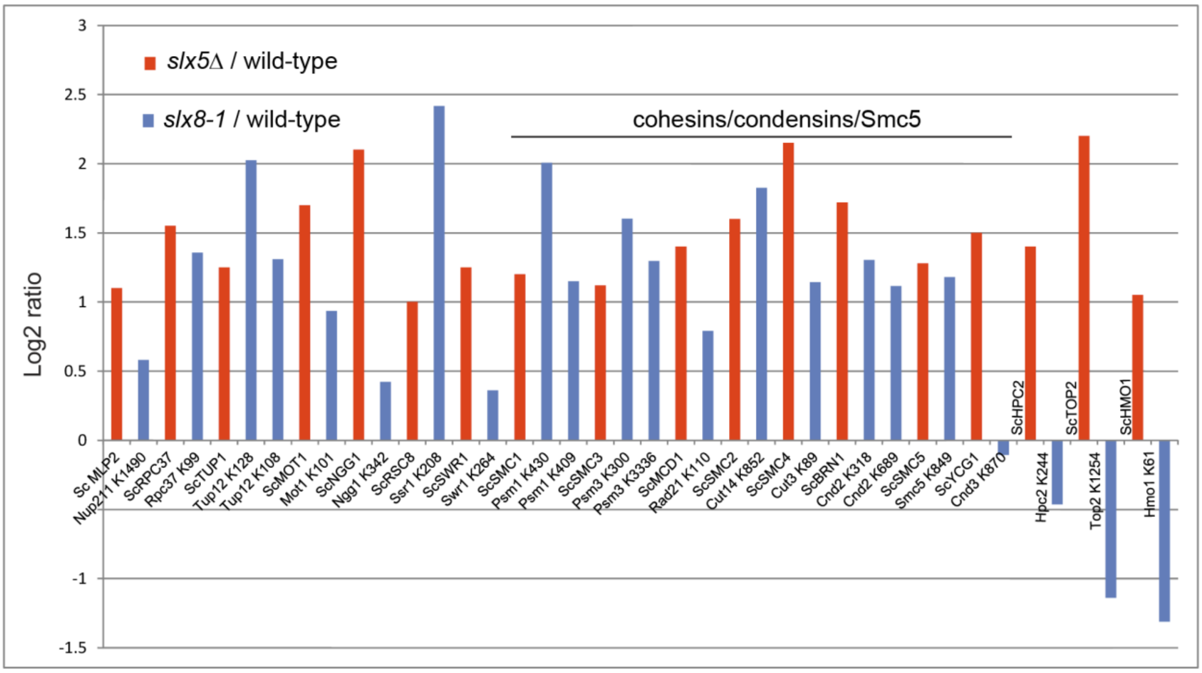

Supplementary Figure 7. Conservation of potential STUbL substrates in *S. pombe* and *S. cerevisiae*. All potential Slx5 substrates identified by Albuquerque *et al*.[7] for which *S. pombe* orthologues could be identified and for which SILAC ratios were obtained in the *slx8-1*/wild-type comparison are displayed. The source for the comparison was Figure 5 in Albuquerque *et al*.[7], displaying 57 factors whose sumoylation level is increased > 2-fold in the *S. cerevisiae slx5Δ* mutant. 46 of these 57 factors have orthologs in *S. pombe*; 50 *S. pombe* orthologs could be identified in YeastMine[2] (2015-05-31 version) due to gene duplications. Sumoylation was not detected for 20 of them in our experiments, in particular septins. Sumoylation was detected for 30 of the 50 *S. pombe* proteins, corresponding to 28 of the 57 *S. cerevisiae* factors. For these 30 *S. pombe* proteins, a total of 51 sumoylation sites were quantified in the *slx8-1*/wild-type SILAC experiment, distributed between 19 proteins corresponding to 18 *S. cerevisiae* proteins. Sumoylation of most of the *S. cerevisiae* orthologs (14/18) was increased in the *S. pombe slx8-1* mutant. There were two exceptions for which decreased sumoylation in *slx8-1* was measured at multiple sites (Top2 and Hmo1) and two exceptions with insignificant decreases (Hpc2 and Cnd3).

Supplementary Table 1. Strains and their genotypes

Strain	Genotype
JK354	*h⁻ ura4-D18 lys3-27 pmt3::His$_6$SUMO int::pJBK111(ura4$^+$)*
JK408	*h⁻ ura4-D18 lys3-27 pmt3::His$_6$SUMO^{L109K} int::pJBK111(ura4$^+$)*
JK414	*h⁻ ura4-D18 lys3-27 pmt3::His$_6$SUMO^{L109K} int::pJBK111(ura4$^+$) ufd1ΔCt$^{213-342}$::natMX6*
JK418	*h⁻ ura4-D18 lys3-27 pmt3::His$_6$SUMO^{L109K} int::pJBK111(ura4$^+$) slx8-1:myc:kanMX6*
JK424	*h⁻ ura4-D18 lys3-27 int::pJBK111(ura4$^+$)*
JK425	*h⁻ ura4-D18 lys3-27 int::pJBK111(ura4$^+$) ufd1ΔCt$^{213-342}$::natMX6*
JK426	*h⁻ ura4-D18 lys3-27 int::pJBK111(ura4$^+$) slx8-1:myc:kanMX6*

Supplementary References

1. Marguerat, S. *et al.* Quantitative analysis of fission yeast transcriptomes and proteomes in proliferating and quiescent cells. *Cell* **151**, 671-83 (2012).
2. Balakrishnan, R. *et al.* YeastMine--an integrated data warehouse for Saccharomyces cerevisiae data as a multipurpose tool-kit. *Database (Oxford)* **2012**, bar062 (2012).
3. Wohlschlegel, J.A., Johnson, E.S., Reed, S.I. & Yates, J.R., 3rd. Global analysis of protein sumoylation in Saccharomyces cerevisiae. *J. Biol. Chem.* **279**, 45662-8 (2004).
4. Hannich, J.T. *et al.* Defining the SUMO-modified proteome by multiple approaches in Saccharomyces cerevisiae. *J. Biol. Chem.* **280**, 4102-10 (2005).
5. Denison, C. *et al.* A proteomic strategy for gaining insights into protein sumoylation in yeast. *Mol. Cell Proteomics* **4**, 246-54 (2005).
6. Panse, V.G., Hardeland, U., Werner, T., Kuster, B. & Hurt, E. A proteome-wide approach identifies sumoylated substrate proteins in yeast. *J. Biol. Chem.* **279**, 41346-51 (2004).
7. Albuquerque, C.P. *et al.* Distinct SUMO ligases cooperate with Esc2 and Slx5 to suppress duplication-mediated genome rearrangements. *PLoS Genet.* **9**, e1003670 (2013).
8. Cox, J. *et al.* Andromeda: a peptide search engine integrated into the MaxQuant environment. *J. Proteome Res.* **10**, 1794-805 (2011).
